# Splicing factor arginine/serine‐rich 8 promotes multiple myeloma malignancy and bone lesion through alternative splicing of CACYBP and exosome‐based cellular communication

**DOI:** 10.1002/ctm2.684

**Published:** 2022-02-20

**Authors:** Yuanjiao Zhang, Xichao Yu, Rongze Sun, Jie Min, Xiaozhu Tang, Zigen Lin, Siyuan Xie, Xinying Li, Shengfeng Lu, Zhidan Tian, Chunyan Gu, Lesheng Teng, Ye Yang

**Affiliations:** ^1^ Nanjing Hospital of Chinese Medicine, Nanjing University of Chinese Medicine, Nanjing, China; ^2^ School of Medicine & Holistic Integrative Medicine Nanjing University of Chinese Medicine Nanjing China; ^3^ School of Life Science Jilin University Changchun China; ^4^ Key Laboratory of A cupuncture and Medicine Research of Ministry of Education Nanjing University of Chinese Medicine Nanjing China; ^5^ Department of Pathology, Nanjing First Hospital Nanjing Medical University Nanjing China

**Keywords:** bone marrow microenvironment, exosomes, multiple myeloma, osteoclast, SFRS8, splicing factor

## Abstract

**Background:**

Multiple myeloma (MM) is a distinctive malignancy of plasma cell within the bone marrow (BM), of which alternative splicing factors play vital roles in the progression. Splicing factor arginine/serine‐rich 8 (SFRS8) is the exclusive factor associated with MM prognosis, however its role in MM remains undefined.

**Methods:**

The analyses of 3‐(4,5)‐dimethylthiahiazo (‐z‐y1)‐3,5‐di‐ phenytetrazoliumromide (MTT) assay, immunohistochemistry, flow cytometry and xenograft model were performed to examine cell proliferation, cell cycle and apoptosis in SFRS8 overexpression or knockdown MM cells in vitro and in vivo. The SFRS8‐regulated alternative splicing events were identified by RNA immunoprecipitation sequencing (RIP‐seq) and validated by RIP‐qPCR and Co‐IP methods. Exosomes were extracted from the supernatant of myeloma cells by ultracentrifugation. Bone lesion was evaluated by TRAP staining in vitro and SCID/NOD‐TIBIA mouse model. A neon electroporation system was utilised to deliver siRNA through exosomes. The effect of siRNA‐loaded exosomes in vivo was evaluated by using a patient‐derived tumor xenograft (PDX) model and SCID/NOD‐TIBIA mouse model.

**Results:**

SFRS8 was significantly upregulated in MM samples and positively associated with poor overall survival (OS) in MM patients. SFRS8 promoted MM cell proliferation in vitro and in vivo. Furthermore, calcyclin binding protein (CACYBP) was identified as the downstream target of SFRS8. Particularly, SFRS8 could reduce CACYBP isoform1 (NM_014412.3) and increase CACYBP isoform2 (NM_001007214.1) by mediating the alternative splicing of CACYBP, thereby altering the ubiquitination degradation of β‐catenin to promote MM progression. In addition, SFRS8 promoted osteoclast differentiation through exosomes in vitro and in vivo. More importantly, exosomal siRNA targeting CACYBP isoform2 inhibited tumour growth in PDX and SCID/NOD‐TIBIA mouse models.

**Conclusion:**

Our findings demonstrate that targeting the SFRS8/CACYBP/β‐catenin axis may be a promising strategy for MM diagnosis and treatment.

## Graphical abstract

Graphic working model illustrates that SFRS8 reduces CACYBP isoform1 (NM_014412.3) and increases CACYBP isoform2 (NM_001007214.1) by mediating the alternative splicing of CACYBP, thereby altering the ubiquitination degradation of β‐catenin and exosome‐based cellular communication to promote MM malignancy and bone lesion.

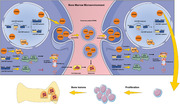

## 1 INTRODUCTION

Multiple myeloma (MM) is a blood system malignancy derived from terminally differentiated plasma cells,^1^ which secrete an excess of monoclonal immunoglobulin protein and originate in the bone marrow (BM).^2^ Currently, the treatment regimens for MM continue to evolve rapidly, in which proteasome inhibitors and immunomodulators are mainly available for clinical application. In addition, multiple treatment algorithms such as monoclonal antibodies, autologous stem cell transplantation and CAR‐T have achieved great success in MM.^3^, ^4^ However, due to the genetic diversity of myeloma cells and their severe dependence on the BM microenvironment, MM patients cannot be cured completely. It is an urgent need for seeking novel promising anti‐MM strategies.

Pre‐mRNA splicing exists in the nucleus of eukaryotic cells, which employs the spliceosome to generate mature mRNA via eliminating intervening introns from primary transcripts and joining exons.^5^ Abnormal alternative splicing produces different variable splicing isoforms, leading to aberrant inactivation of tumour suppressors or activation of oncogenes and cancer pathways.^6^ Abnormal alterations of oncogenic splicing events or their upstream splicing regulators contribute to the oncogenesis and development of various cancers, especially in hematological cancers.^7^ The exploration of novel therapies on manipulation of splicing is of great importance.^8^

Our preliminary study identified that splicing factor arginine/serine‐rich 8 (SFRS8) was highly expressed in MM patients from the microarray data of MM patient cohorts. SFRS8 encodes for an serine/arginine (SR)‐like protein that contains an arginine/serine (RS)‐domain, which is a putative splicing factor.^9^, ^10^ It is known that proteins containing RS‐domains regulate RNA splicing, transcript elongation and stability, export of nuclear, cleavage of miRNA and stability of genome.^11^, ^12^ SFRS8 serves as a splicing factor regulating the splicing of CD45 in atopy and asthma, while CD45 is a crucial molecule in the activation process of T cells.^13^ To date, there are few reports focusing on the function of SFRS8 in cancers, especially in MM.

The present study demonstrated the correlations between SFRS8 expression and the outcomes and pathological characteristics of MM patients. We aimed to determine the promising downstream target of SFRS8 to elucidate the potential mechanism of SFRS8 promoting MM progression.

## 2 MATERIALS AND METHODS

### 2.1 Gene expression profiling

The gene expression profiling (GEP) of MM patients were obtained from the GEO database as previously described.^14^, ^15^ The information of mRNA sequencing, kyoto encyclopedia of genes and genomes (KEGG) pathway enrichment analysis and gene ontology (GO) function significance enrichment analysis was in supporting information.

### 2.2 Antibodies and reagents

The primary antibodies used in this study were at the dilutions of 1:1000 as follows: SFRS8 (24705‐1‐AP, ProteinTech Group, China; ab72044, Abcam, UK); poly ADP‐ribose polymerase (PARP) (9542S, Cell Signaling Technology, USA); β‐catenin (51067‐1‐AP, ProteinTech Group, China); β‐actin (4970S, Cell Signaling Technology, USA); Alix (2171S, Cell Signaling Technology, USA); CD9 (13174S, Cell Signaling Technology, USA); Calnexin (10427‐2‐AP, ProteinTech Group, China); Ubiquitin (10201‐2‐AP, ProteinTech Group, China); HA (51064‐2‐AP, ProteinTech Group, China); DYKDDDDK (98533S, Cell Signaling Technology, USA). The anti‐DYKDDDDK antibody (101274‐mm05t, Sino Biological, China) was at the 1:50 dilution, and Goat pab to Ms IgG (FITC) (ab6785, Abcam, UK) was at the 1:200 dilution. The second antibodies Goat anti‐Rabbit IgG(H+L) HRP (FMS‐Rb01, Fcmacs) and goat anti‐Mouse IgG (H+L) HRP (S0002, Affinity) were in 5 000 diluted concentrations.

Doxycycline (DOX), rabbit IgG (a7016) and mouse IgG (a7028) were purchased from the Beyotime (Shanghai, China). Puromycin was obtained from Merck KGaA (Darmstadt, Germany). Diphenyltetrazolium Bromide (MTT) was purchased from Solarbio (Shanghai, China). Methyl 3‐{[(4‐methylphenyl) sulfonyl] amino}benzoate (MSAB) was purchased from Selleck (Shanghai, China). GW4869 was purchased from MedChemExpress (Monmouth Junction, NJ, USA).

### 2.3 Cell lines and cell culture

Human MM cell lines ARP1 and CAG, and peripheral blood mononuclear cells (PBMCs) were cultured in RPMI‐1640 (Biological Industries, Israel). HEK293 and RAW264.7 cells were cultured in DMEM (Thermo Fisher Scientific, USA). Culture medium was added with fetal bovine serum (10%, Gibco, USA), penicillin (100 U/ml, HyClone, USA) and streptomycin (100 μg/ml, HyClone, USA), which was changed every 2–3 days. All cells were cultured in 100 mm dishes at 37°C in 5% CO_2_ incubator.

### 2.4 Plasmids and cell transfection

Plasmids containing human SFRS8 cDNA and SFRS8 shRNA cassettes were provided by TranSheepBio (Shanghai, China). SFRS8 coding sequence was cloned into CD513B1 vector with green fluorescence and Flag tags; SFRS8‐targeting shRNA was inserted into pTRIPZ vector, which was controlled by a DOX‐inducible promoter. Lentivirus packaging and transfection steps were performed as previously described.^16^ The expression vector and packaging vector (PLP1, PLP2, VSVG) were co‐transfected into HEK293 cells according to the Lipofectamine Transfection Reagent (YEASEN, Shanghai) method to obtain lentivirus. After 48 h, the virus supernatant was collected, concentrated and stored at −80°C. The transfected cells were screened by puromycin with high transduction efficiency.

### 2.5 Cell proliferation, cell cycle and apoptosis assays

The 3‐(4,5)‐dimethylthiahiazo (‐z‐y1)‐3,5‐di‐ phenytetrazoliumromide (MTT) method was performed to test the proliferation rate and cell viability for 24, 48 and 72 h, respectively. The absorbance at 570 nm was measured to calculate the number of viable cells.

Flow cytometry (Merck Millipore, Darmstadt, Germany) was applied to detect cell cycle and apoptosis, and the methods were performed as described previously.^17^

### 2.6 Co‐immunoprecipitation

According to the manufacturer's instructions, the Pierce Direct Magnetic IP/CO immunoprecipitation (Co‐IP) kit (Thermo Scientific) was utilised for Co‐IP assay.

### 2.7 Quantitative PCR

Sequences of the primers and small interfering RNAs (siRNAs) were presented in Table S1 and S2. Total RNA was isolated from MM cells by using Trizol reagent (YEASEN, Shanghai). Reverse transcription kit (YEASEN, Shanghai) was used to synthesise complementary DNA. Quantitative PCR (qPCR) were performed with SYBR Green master Mix (YEASEN, Shanghai) using GAPDH as loading control. qPCR was performed by Analytikjena qPCR soft 4.0 (Germany). The qPCR reaction program was conducted as follows: predenaturation temperature is 95°C, 3 min; denaturation temperature is 95°C, 10 s; annealing temperature is 60°C, 59 s; a total of 40 cycles. We calculated the relative expression levels of target genes by the 2−ΔΔCT method and graphed as fold change relative to control.

### 2.8 RNA immunoprecipitation sequencing

The RNA immunoprecipitation sequencing (RIP‐seq) was performed on the Illumina sequencing platform in Genedenovo Biotechnology Co., Ltd (Guangzhou, China).

### 2.9 Tartrate‐resistant acid phosphatase activity staining

RAW264 cells were seeded in a 24‐well plate at the density of 3,000 cells/well. Supplemented with recombinant murine sRANKL (50 ng/ml, Peprotech, USA) and M‐CSF (10 ng/ml, Peprotech, USA), the culture medium was changed every other day. After 6 days, the Leukocyte‐Tartrate‐resistant acid phosphatase (TRAP) kit (Sigma–Aldrich; Merck KGaA) was utilised to stain the cells to detect the TRAP activity. Finally, the samples were stained with 1% aqueous Fast Green FCF for 1 min.^18^

Ficoll‐Paque (Salarbio) density gradient centrifugation was used to extract PBMCs. PBMCs were seeded in a 24‐well plate at the density of 1 × 10^6^ cells/well. The culture conditions were the same as used in RAW264 cells. After 15 days, the degree of osteoclast differentiation was determined by detecting the TRAP activity of the cells.

### 2.10 Exosome isolation and confirmation

The supernatant of ARP1 WT cells was collected and centrifuged using the following procedure: 300×*g* for 10 min, 2 000×*g* for 10 min, 10 000×*g* for 30 min to remove floating cells and debris. The remaining supernatant was centrifuged in an ultracentrifuge at 100,000×*g* for 70 min. Then, we washed the collected precipitate with phosphate buffered saline (PBS), and centrifuged again at 100,000×*g* for 70 min to collect the precipitate, resuspended in 200 μl PBS, and store at −80°C. The morphology was identified by A JEM‐2100 transmission electron microscope (JEOL, Tokyo, Japan). The markers Alix and CD9, and negative maker calnexin were detected by western blot (WB) analysis.

### 2.11 Immunofluorescent staining and confocal microscopy

Immunofluorescent staining was performed as described previously.^19^ A confocal microscope (TCS SP8, Leica, Germany) was used to capture images.

### 2.12 Immunohistochemistry analysis

Immunostaining was performed on paraffin tissue sections. The main process was as follows: the samples on the slides were incubated with the primary antibody at 4°C overnight. Afterwards, the secondary antibody was applied and kept at 37°C for 45 min, followed by dropping SABC at 37°C for 30 min. The samples were coloured brown with incubation of a diaminobenzidine solution and finally followed by light counterstaining with hematoxylin.

### 2.13 Multiple myeloma mouse models

#### 2.13.1 Multiple myeloma xenograft model

We used 6 to 8‐week old SCID/NOD mice to establish a MM xenograft model. 1 × 10^6^ SFRS8‐knockdown (SFRS8‐KD) cells were injected subcutaneously into the left abdominal flanks of the mice. At day 10 after tumour implantation, doxycycline was added to drinking water containing 5% sucrose at the final concentration of 2 mg/ml to induce knockdowning SFRS8. The diameters of the tumours were measured with a caliper every other day. When the diameter of the xenografted tumour reached 20 mm, we sacrificed the mice and collected, weighed the tumours, and then photographed tumour tissues.^20^, ^21^

#### 2.13.2 SCID/NOD‐TIBIA mouse model

1 × 10^7^/10 μl ARP1 WT or SFRS8‐overexpression (SFRS8‐OE) cells were injected into the BM cavity of the tibias of the 6 to 8‐week old SCID/NOD mice. In order to check the osteolysis, the bone density and volume of the tibia were analysed by micro‐computed tomography (μCT; SkyScan 1176, Bruker microCT, Germany).^22^

#### 2.13.3 PDX model

The patient‐derived tumor xenograft (PDX_ model was generated by using the biopsy samples collected from an extramedullary tumour subcutaneously under the head skin of a MM patient at the Department of Hematology, the First Affiliated Hospital of Nanjing Medical University. According to the method reported by Zhou et al.,^23^ the tumour slices were transplanted subcutaneously in 4 to 6‐week old male SCID/NOD mice (*n* = 6) under pentobarbital anaesthesia. The tumours were harvested once their sizes reached 500 mm^3^, then the tumour tissues were divided into 2.5 × 2.5 × 2.5 mm^3^ pieces and subcutaneously implanted again. After this process was repeated for three times and the tumour size reached 100–150 mm^3^, the mice were randomly divided into control and treatment groups, which were injected with PBS or calcyclin binding protein (CACYBP) siRNA‐loaded exosomes (Exo) every three days.

All animal works were performed in accordance with the Government‐published recommendations for the Care and Use of Laboratory Animals, and Guidelines of Institutional Ethics Review Boards of Nanjing University of Chinese Medicine (Ethics Registration no. 201905A003).

### 2.14 Statistical analysis

All data were expressed as the mean ± standard deviation. Two‐tailed Student's *t*‐test (two groups) and one‐way analysis of variance for multiple comparisons were used to determine significance between experimental groups. The Kaplan–Meier method and Log‐rank test were used to determine the survival rate of MM patients. *p* < 0.05 (*), *p* < 0.01 (**) and *p* < 0.001 (***) were indicated that statistically significant differences.

## 3 RESULTS

### 3.1 Elevated SFRS8 is associated with poor MM patient survival and promotes MM cell proliferation in vitro and in vivo

**FIGURE 1:**
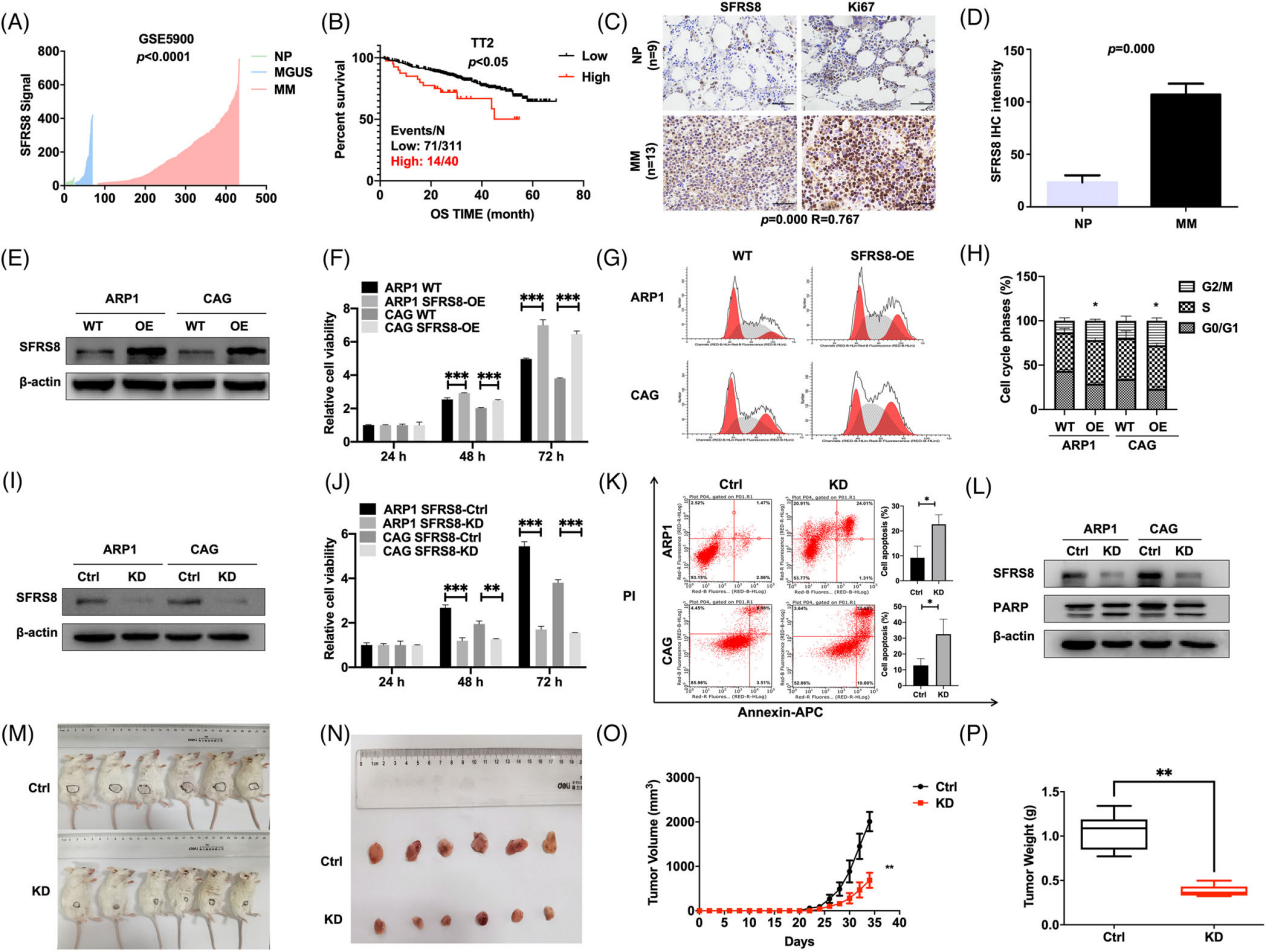
Elevated splicing factor arginine/serine‐rich 8 (SFRS8) is relevant to poor survival and promotes multiple myeloma (MM) proliferation in vitro and in vivo. (A) SFRS8 mRNA levels were significantly elevated in MM patients of GSE5900 dataset. The signal level of SFRS8 was shown on the *y*‐axis. The groups of healthy donors with normal bone marrow (BM) plasma cells (normal plasma, NP, *n* = 22), monoclonal gammopathy of undetermined significance (MGUS, *n* = 44), and MM (*n* = 351) were displayed on the *x*‐axis respectively. (B) Increased SFRS8 was significantly correlated with poor MM overall survival (OS) in TT2 cohort. (C) Representative immunohistochemistry (IHC) staining of primary MM (*n* = 13) and NP samples (*n* = 9). Scale bar: 50 μm. (D) Statistical analysis of SFRS8 IHC staining intensity between NP and MM groups. (E) Western blot (WB) examined SFRS8 expression in WT and SFRS8‐OE MM cells. (F) The proliferation capacity in WT and SFRS8‐OE MM cells was tested by MTT assay. (G,H) Flow cytometry revealed that the proportion of G2/M phase was significantly increased in SFRS8‐OE cells compared with WT cells. (I) WB verified the expression of SFRS8 upon transfection with SFRS8‐targeting shRNAs. (J) MTT assay tested the proliferation rate of SFRS8‐KD and Ctrl MM cells. (K) The apoptosis of SFRS8‐KD and Ctrl MM cells was determined by flow cytometry. (L) WB showed the protein levels of SFRS8 and PARP in SFRS8‐KD and Ctrl MM cells. (M) Photographic images of xenograft mice were captured at day 34. (N) Schematic images of xenografts from SCID/NOD mice. (O) Time course of tumour growth in SCID/NOD mice. (P) Mean tumour weight in SCID/NOD mice. SFRS8‐Ctrl and SFRS8‐KD cells were injected subcutaneously into SCID/NOD mice. The data are expressed as mean ± SD. *p < 0.05, **p < 0.01, ***p < 0.001. WT: wild type; OE: overexpression; Ctrl: control; KD: knockdown

The MM GEP cohorts were analysed to determine the expression of SFRS8 in MM. The data showed that SFRS8 expression in patients of monoclonal gammopathy of undetermined significance (MGUS, *n* = 44) and MM (*n* = 351) was obviously increased compared with normal plasma (NP, *n* = 22) (*p* < 0.0001) (Figure 1A). Moreover, upregulation of SFRS8 was significantly associated with worse outcome in total therapy 2 (TT2) cohort (*p* < 0.05) (Figure 1B). In agreement with above results, immunohistochemistry (IHC) assay corroborated that SFRS8 was strongly expressed in MM primary samples relative to the normal control tissues. In addition, Ki67, a key marker of cell proliferation, was positively correlated with SFRS8 expression (*p* = 0.000, *R* = 0.767) (Figure 1C, D). These clinical evidences confirmed the correlation of SFRS8 expression with abnormal cell proliferation and poor prognosis of MM patients.

We further explored the functions of SFRS8 in MM cell lines. Initially, we stably overexpressed SFRS8 in ARP1 and CAG cells confirmed by WB analysis (Figure 1E). The proliferation capacity of ARP1 and CAG cells was remarkably increased upon upregulating SFRS8 expression (*p* < 0.001) (Figure 1F). Cell cycle analysis demonstrated an evidently higher G2/M phase proportion in SFRS8‐OE cells than WT cells (*p* < 0.05) (Figure 1G, H). Conversely, we transfected MM cells with SFRS8 lentiviral shRNA particles, and the knockdown efficiency was validated by WB analysis (Figure 1I). The cell proliferation was significantly decreased in SFRS8‐KD cells compared with control cells (*p* < 0.001) (Figure 1J). The flow cytometry experiment proved that inducible downregulation of SFRS8 promoted cell apoptosis (*p* < 0.05) (Figure 1K). Additionally, we developed WB analysis to confirm an increased expression of PARP in SFRS8‐KD cells (Figure 1L).

To extend these findings in vivo, we injected SFRS8‐Ctrl and SFRS8‐KD cells subcutaneously into SCID/NOD mice. As illustrated in Figure 1M, the tumours formed by SFRS8‐KD cells were visibly smaller than SFRS8‐Ctrl counterparts after 4 weeks (Figure 1M,N). The tumour growth curve showed that the average volume of SFRS8‐KD tumours significantly lagged behind the corresponding control tumours (*p* < 0.01) (Figure 1O). The analysis of tumour weight was consistent with the data of tumour volume (*p* < 0.01) (Figure 1P). The expression of SFRS8 protein was decreased in SFRS8‐KD tumours compared with SFRS8‐Ctrl tumours (Figure S1).

### 3.2 SFRS8 activates alternative splicing of CACYBP mRNA in MM cells

**FIGURE 2:**
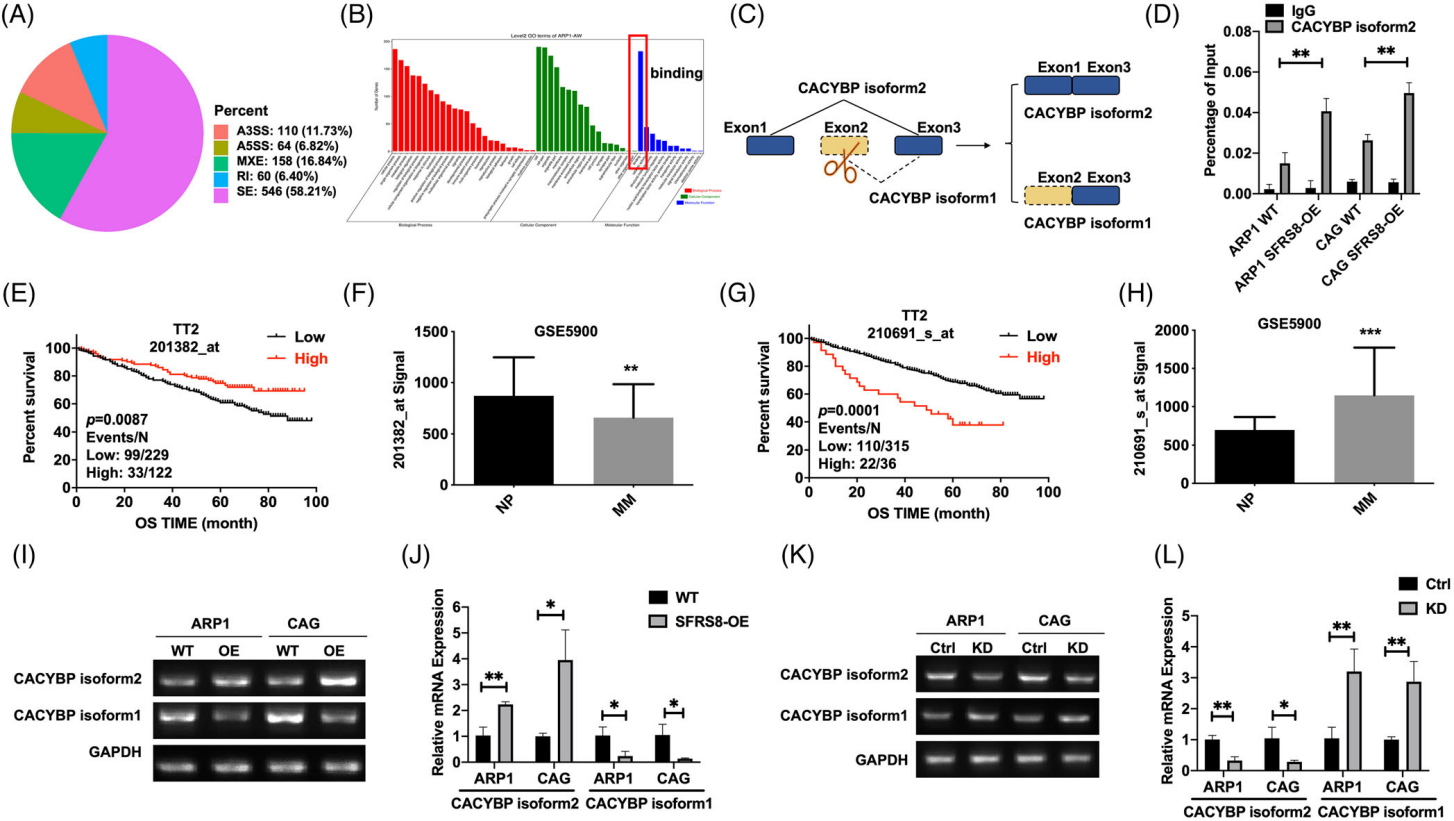
Splicing factor arginine/serine‐rich 8 (SFRS8) activates alternative splicing of CACYBP mRNA. (A) RIP‐seq assay showed the numbers of alternative splicing events illustrated in each category. A3SS: alternative 3′ splice site; A5SS: alternative 5′ splice site; MXE: mutually exclusive exon; RI: retained intron; SE: skipped exon. (B) GO molecular function enrichment of SFRS8‐regulated alternative splicing genes. (C) The pattern diagram of SFRS8 spliced CACYBP through exon skipping. (D) RIP‐qPCR indicated overexpressed SFRS8 directly upregulated the expression of CACYBP isoform2. (E–H) Survival proportions of MM patients corresponding to different probes of CACYBP in TT2 cohort. (E,F) 201382_at predicted the favourable survival time of MM patients, (G,H) while other probes represented opposite survival time. (I,J) The expression of CACYBP isoform2 was increased, while the expression of CACYBP isoform1 was decreased in SFRS8‐OE MM cells. (K,L) Decreased SFRS8 significantly upregulated the expression of CACYBP isoform1 and downregulated the expression of CACYBP isoform2. The data are expressed as mean ± SD. *p < 0.05, **p < 0.01, ***p < 0.001

In order to further reveal the regulatory mechanism of SFRS8 in MM, we utilised RIP‐seq assay using SFRS8 antibody as a bait in MM cells and defined a total of 938 alternative splicing events with significant differences. Among these alternative splicing events, exon skipping events were accounted for 58.21% (546/938) (Figure 2A). The data of GO function significance enrichment analysis indicated that SFRS8‐regulated alternative splicing events were mainly related with cell composition, binding and other functions (Figure 2B). We next combined the analyses of RIP‐seq and MM GEP cohorts to screen the downstream targets of SFRS8, which were spliced upon SFRS8 modification and associated with MM progression. Based on this strategy, CACYBP was distinguished as the top‐ranked downstream target gene according to the significant increment of CACYBP exon 2 skipping splice variants expression post SFRS8 induction. There are two splicing isoforms CACYBP isoform1 (NM_014412.3) and CACYBP isoform2 (NM_001007214.1) in MM. As shown in Figure 2C, SFRS8 spliced CACYBP through exon skipping. We further adopted RNA immunoprecipitation analysis to assess the alternative splicing‐dependent effect of SFRS8 on CACYBP mRNA. Compared with negative IgG, SFRS8 directly bound to endogenous CACYBP isoform2 and promoted its expression in WT and SFRS8‐OE cells (*p* < 0.01) (Figure 2D). The probe 201382_at designed based on CACYBP isoform1 was associated with favourable OS in TT2 cohort (*p* = 0.0087) (Figure 2E). The MM GEP data showed that CACYBP isoform1 expression in MM patients (MM, *n* = 351) was obviously decreased compared with NP cells (*n* = 22) (*p* < 0.001) (Figure 2F). In contrast, other probes like the probe 210691_at predicted worse survival of MM patients and increased significantly in MM (*p* = 0.0001, *p* < 0.001) (Figure 2G,H).

We next detected the expression levels of the two isoforms in SFRS8‐OE and SFRS8‐KD cells by the qPCR method. The expression of CACYBP isoform2 was significantly increased in SFRS8‐OE cells and decreased in SFRS8‐KD cells compared with control cells (*p* < 0.05, *p* < 0.01) (Figure 2I–L), however, CACYBP isoform1 exhibited a diametrically opposite trend relative to CACYBP isoform2 (*p* < 0.05, *p* < 0.01) (Figure 2I–L). We also performed the qPCR assay to examine the mRNA expressions of SFRS8, CACYBP isoform2 and CACYBP isoform1 in tumour samples. The mRNA expressions of SFRS8 and CACYBP isoform2 were decreased, while the mRNA expression of CACYBP isoform1 was increased in SFRS8‐KD tumours compared with SFRS8‐Ctrl tumours (*p* < 0.001) (Figure S2).

### 3.3 Aberrant splicing of CACYBP contributes to MM cell proliferation and decreases β‐catenin ubiquitination in vitro

**FIGURE 3:**
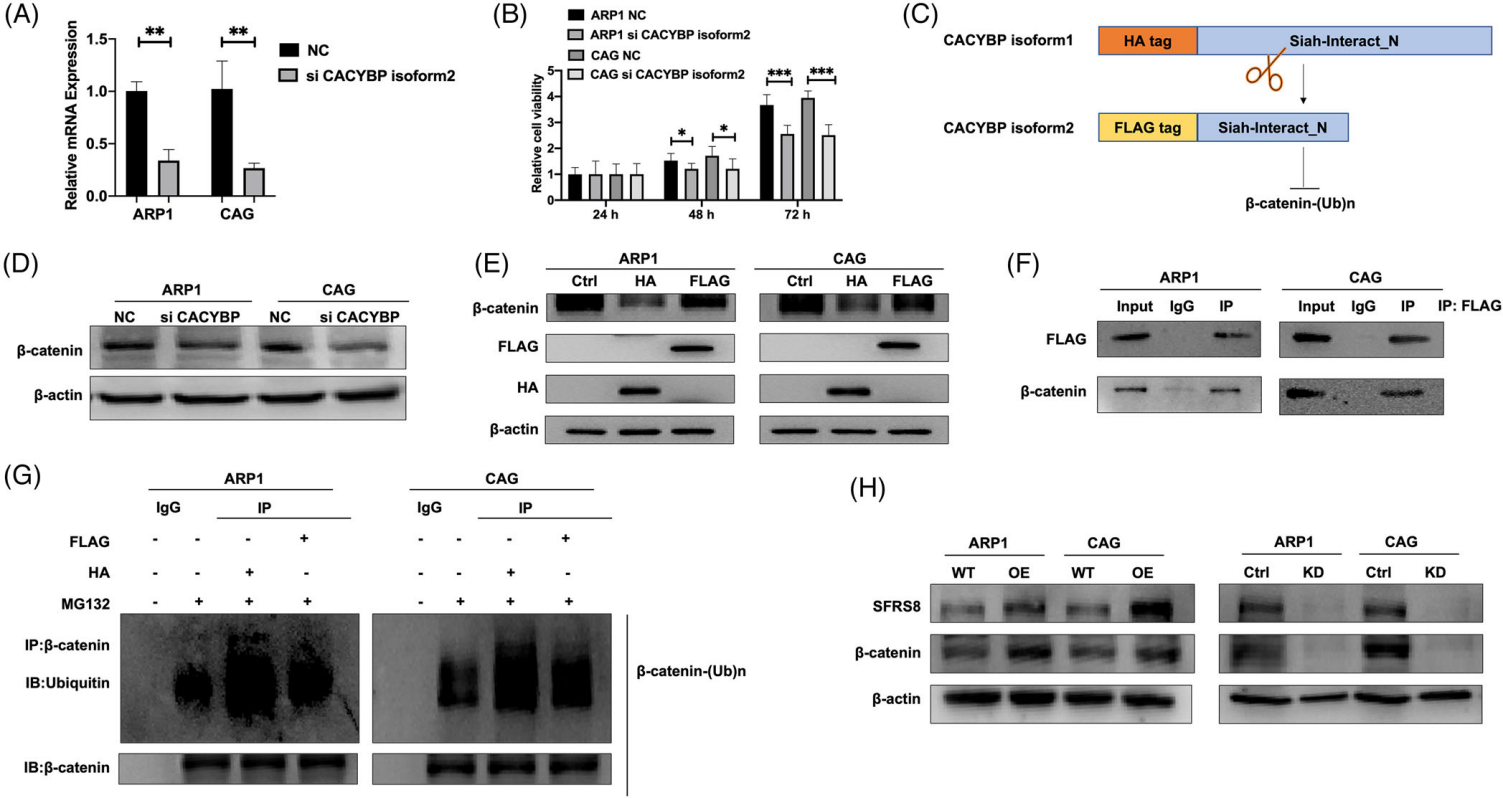
Aberrant splicing of CACYBP evokes multiple myeloma (MM) cell proliferation and decreases β‐catenin ubiquitination in vitro. (A) CACYBP isoform2 was decreased upon using specific siRNAs to target CACYBP isoform2 determined by qPCR. (B) MTT tested the proliferation of ARP1 and CAG cells upon knockdown of CACYBP isoform2. (C) Graphic illustration of CACYBP isoform1‐OE plasmid with HA tag and CACYBP isoform2‐OE plasmid with FLAG tag, respectively. (D) Western blot (WB) detected the expression of β‐catenin in CACYBP isoform2‐KD cells. (E) WB examined the expression of β‐catenin in FLAG‐tagged CACYBP isoform2 and HA‐tagged CACYBP isoform1 cells. (F) Co‐IP experiment showed an interaction between FLAG‐tagged CACYBP and β‐catenin in MM cells. (G) The ubiquitination and degradation of β‐catenin upon transfection of CACYBP with FLAG and HA tags, respectively, followed by the treatment of MG132 for 12 h. (H) WB detected β‐catenin expression in SFRS8‐OE and SFRS8‐KD cells. The data are expressed as mean ± SD. *p < 0.05, **p < 0.01. Ctrl: control; KD: knockdown

We further examined the effect of splicing variants of CACYBP on MM cell proliferation. CACYBP isoform2 interfered with a specific siRNA was decreased significantly (*p* < 0.01) (Figure 3A). MTT experiment demonstrated that silencing CACYBP isoform2 evidently hampered MM cell proliferation (*p* < 0.05, *p* < 0.001) (Figure 3B). CACYBP is known as a Siah‐1 ligand,^24^ and Siah‐1 polyubiquitinates β‐catenin to facilitate its degradation.^25^ β‐catenin is overexpressed in malignant plasma cells and meditates the critical events in the development of MM.^26^ It was conceivable that the alternative splicing events affecting CACYBP could regulate the degradation of β‐catenin (Figure 3C). The WB assay confirmed that β‐catenin expression was decreased in siCACYBP isoform2 cells relative to negative control (NC) cells (Figure 3D). Furthermore, we constructed the CACYBP isoform1 OE plasmid with HA tag and CACYBP isoform2 OE plasmid with FLAG tag to explore the molecular functions, respectively. The FLAG‐tagged isoform2 (CACYBP isoform2) promoted the expression of β‐catenin, while the HA‐tagged isoform1 (CACYBP isoform1) exerted the converse action (Figure 3E). The Co‐IP experiment indicated that CACYBP isoform2 and β‐catenin directly interacted with each other (Figure 3F). To determine the half‐life of β‐catenin affected by CACYBP isoforms, ARP1 and CAG cells were treated with 50 μg/ml cycloheximide (CHX, MedChemExpress), a protein synthesis inhibitor, and cultured for 0, 2, 4, 6, 8, 12 h. The β‐catenin protein levels were detected at different time points. It was observed that the half‐life of β‐catenin protein was longer in FLAG‐tagged CACYBP isoform2 cells than HA‐tagged CACYBP isoform1 cells (Figure S3). And further ubiquitination experiments indicated that FLAG‐tagged CACYBP isoform2 resulted in less ubiquitination and degradation of β‐catenin compared with HA‐tagged CACYBP isoform1 (Figure 3G). In addition, the expression of β‐catenin was elevated in SFRS8‐OE cells and reduced in SFRS8‐KD cells (Figure 3H). The above results suggested that elevated SFRS8 mediated the increment of CACYBP isoform2 leading to a decrease in the ubiquitination and degradation of β‐catenin.

### 3.4 SFRS8 induces osteoclast differentiation in the bone marrow microenvironment through exosomes and promotes bone destruction in the SCID/NOD‐TIBIA mouse model

**FIGURE 4:**
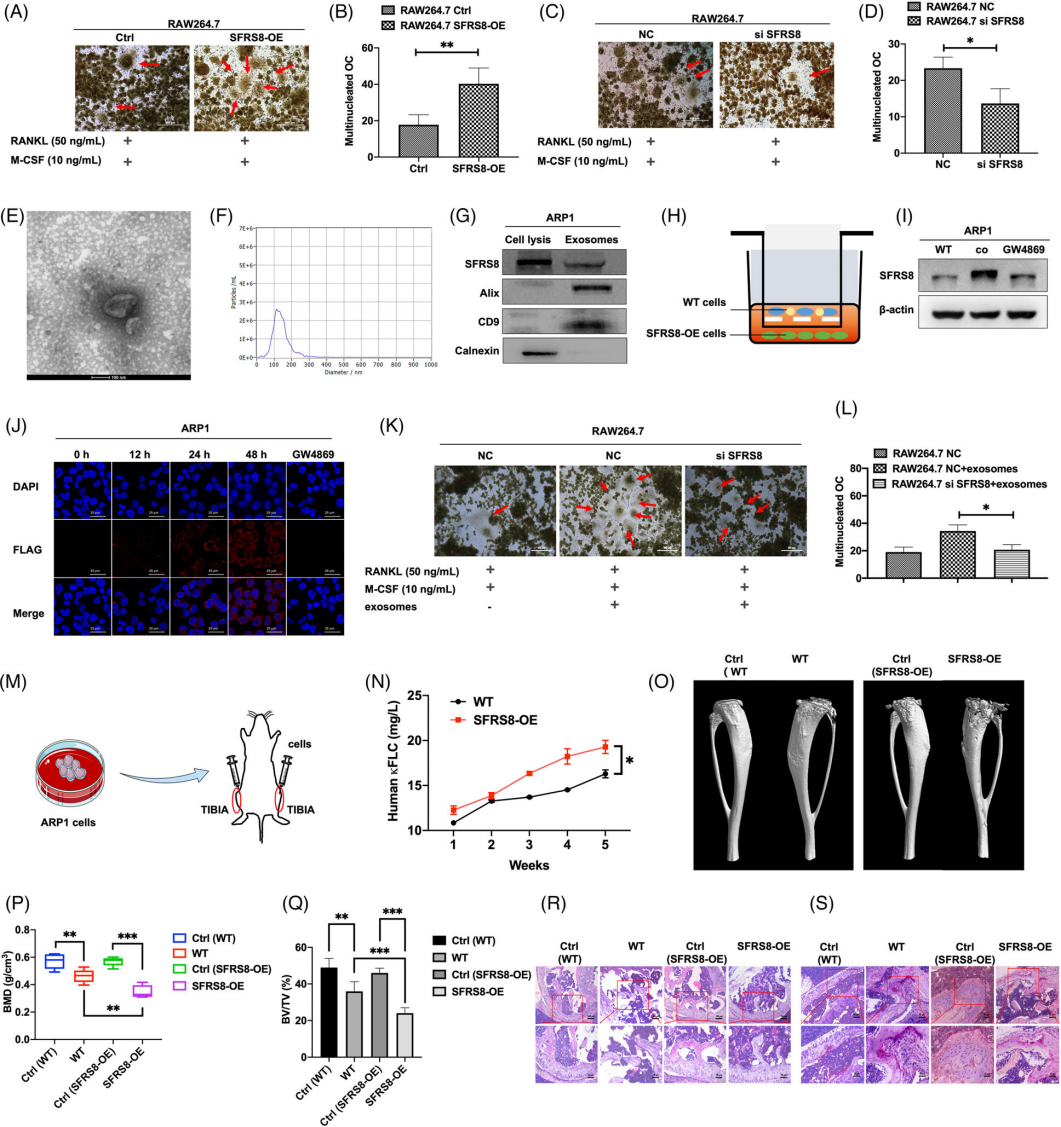
Exosomal Splicing factor arginine/serine‐rich 8 (SFRS8) induces osteoclast differentiation in the bone marrow (BM) microenvironment and overexpression of SFRS8 aggravates bone destruction in SCID/NOD‐TIBIA mouse model in vivo. (A) TRAP activity results revealed that elevated SFRS8 promoted the differentiation of RAW264.7 cells into osteoclasts. Scale bar: 500 μm. (B) Statistics on the number of osteoclasts in RAW264.7 Ctrl and SFRS8‐OE cells. (C) SFRS8 siRNA (si SFRS8) inhibited the differentiation of RAW264.7 cells into osteoclasts detected by TRAP activity assay. Scale bar: 500 μm. (D) Statistical analysis on the number of osteoclasts upon silencing of SFRS8. (E) The biological characteristics of exosomes were detected by transmission electron microscopy. Scale bar: 100 nm. (F) NTA analysis of exosomes. (G) Western blot analysis of SFRS8, calnexin and Alix &CD9. (H) Graphic illustration of co‐cultured ARP1 WT cells with ARP1 SFRS8‐OE cells using transwell assay. (I) WB assay showed the expression of SFRS8 in supernatant of the cocultured cells upon the treatment of GW4869. (J) ARP1 WT cells time‐dependently gained increased SFRS8 expression detected by confocal microscopy. Scale bar: 25 μm. (K) TRAP staining experiments revealed that exosomes promoted osteoclast differentiation. Scale bar: 500 μM. (L) Statistical analysis on the number of osteoclasts. (M) Graphic illustration of SCID/NOD‐TIBIA mouse model. (N) Human κFLC levels in the mouse serum measured by ELISA. (O) Representative microCT images of bones in WT and SFRS8‐OE groups. (P) Bone mineral density (BMD) of SCID/NOD‐TIBIA mice in WT and SFRS8‐OE groups. (Q) Bone volume (BV/TV) of SCID/NOD‐TIBIA mice in WT and SFRS8‐OE groups. (R) H&E staining of histological sections of bones in WT and SFRS8‐OE groups. Scale bar: 100 μM, 50 μM. (S) TRAP staining of histological sections of bones in WT and SFRS8‐OE groups. Scale bar: 50 μM, 25 μM. The data are expressed as mean ± SD. *p < 0.05, **p < 0.01, ***p < 0.001. Ctrl: control; NC: negative control

In our previous studies, it has been evidenced that the BM microenvironment supports oncogenic growth of MM cells.^16^, ^27^ The osteolytic lesions caused by the malignant proliferation and differentiation of osteoclasts (OCs) in the BM microenvironment aggravate the situation and reduce the survival of MM patients.^28^, ^29^ Here, we accessed the association of SFRS8 with osteoclast differentiation by using pre‐osteoclast RAW264.7 cells transfected with SFRS8 plasmid. TRAP activity results showed that increased SFRS8 promoted the differentiation of RAW264.7 cells into OCs with the treatment of recombinant murine sRANKL (50 ng/ml) and M‐CSF (10 ng/ml) compared with WT cells (*p* < 0.01) (Figure 4A,B). Conversely, silencing SFRS8 expression significantly reduced the formation of osteoclast (*p* < 0.05) (Figure 4C,D).

As the Exos in the BM microenvironment possess the high capacity to trigger osteolysis,^30^ we continued to investigate whether SFRS8 could promote osteoclast differentiation through Exos. Exos were extracted from ARP1 WT cells by using ultracentrifugation, followed by utilising transmission electron microscopy to detect Exos with a morphological size of 30–150 nm and a cup‐plate‐like structure (Figure 4E). In addition, nanoparticle tracking analysis (NTA) proved that the Exos were around 100 nm in diameter (Figure 4F). The exosomal markers Alix and CD9 were determined by WB analysis, while the endoplasmic reticulum protein calnexin could not be detected in Exos (Figure 4G). We also cocultured ARP1 WT cells with ARP1 SFRS8‐OE cells by hanging cell culture inserts (Figure 4H) and found that the cocultured WT cells overexpressed SFRS8 compared with WT cells and GW4869 treated cells (Figure 4I). GW4869 is a well‐recognised Exo inhibitor reducing Exos release.^31^ IF staining for FLAG and DAPI revealed that the expression of SFRS8 was increased in a time‐dependent manner in cocultured WT cells compared with GW4869 treated cells (Figure 4J). Above analyses indicated that SFRS8 could not migrate into the cocultured cells upon GW4869 treatment, suggesting that SFRS8 secreted by MM cells might affect the surrounding cells through Exos. Consistently, the rescue experiment of osteoclast differentiation also confirmed that the osteoclast differentiation was augmented with the existence of Exos, while SFRS8 was normally expressed (*p* < 0.01) (Figure 4K,L). Taken together, these findings support that SFRS8 is secreted by MM cells through Exos to promote osteoclast differentiation, thereby favouring MM cell proliferation.

To extend the exploration on the functions of SFRS8 in vivo, we utilised a SCID/NOD‐TIBIA mouse model (Figure 4M). ARP1 WT and SFRS8‐OE cells were injected into the BM cavity of tibias in SCID/NOD mice, respectively (Figure S4). Blood drawn was performed weekly, and the tumour burden was determined by blood κFLC levels via an ELISA κFLC detection kit (YIFEIXUE BIO TECH, China). Osteolysis can be reflected by the serum levels of κFLC, a biomarker of MM tumor burden.^32^, ^33^ The levels of κFLC were increased earlier and more quickly in SFRS8‐OE group than WT group (*p* < 0.05) (Figure 4N). We also used μCT to analyse the bone density and volume of the tibia. MicroCT imaging of the bones indicated that bone damage was significantly severe in SFRS8‐OE group compared with the WT group at the end of the experiment (Figure 4O). Bone mineral density (BMD) and bone volume (BV/TV) were remarkably decreased in the SFRS8‐OE group compared with the WT group (*p* < 0.01, *p* < 0.001) (Figure 4P,Q). In addition, the analyses of H&E staining and TRAP staining illustrated that the bone samples in the SFRS8‐OE group showed a higher density of tumour cells with intact structures (Figure 4R), as well as increased numbers of OCs (Figure 4S) than controls.

### 3.5 Wnt/β‐catenin pathway is involved in the regulation of SFRS8 on osteoclast differentiation

**FIGURE 5:**
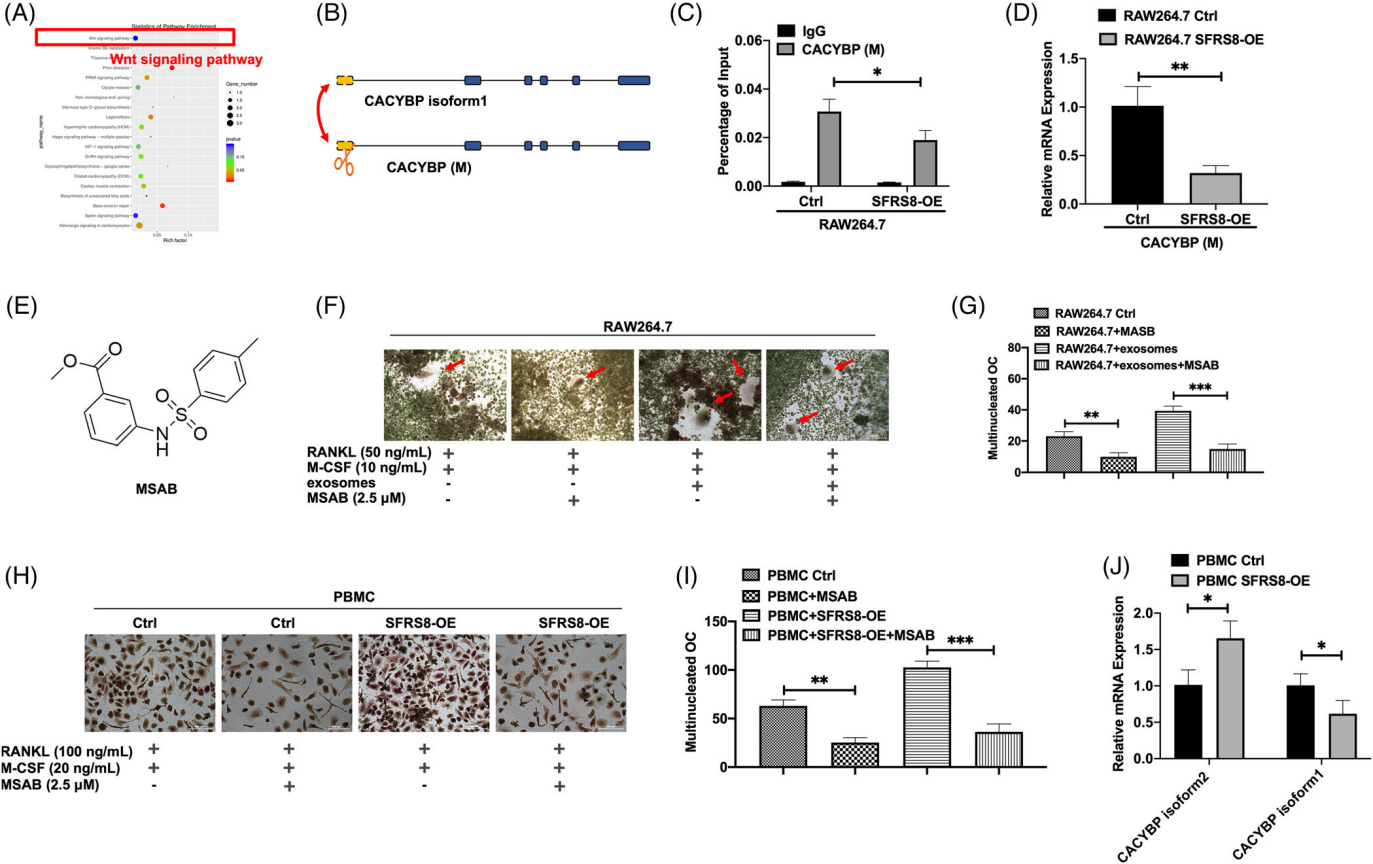
The regulation of splicing factor arginine/serine‐rich 8 (SFRS8) on osteoclast differentiation is mediated by Wnt/β‐catenin pathway. (A) KEGG pathway enrichment analysis for SFRS8 siRNA (si SFRS8) vs. NC was shown as scatter plots. The *Y*‐axis represented the 20 enriched pathways (based on a corrected *p*‐value) and the *X*‐axis represented the richness factor reflecting the proportion of differential expressed genes (DEG) in any given pathway. The numbers of DEGs in the pathway were indicated by the circle area. The circle colour represented the range of the corrected *p*‐values. (B) Graphic illustration of CACYBP isoform1 and mouse CACYBP. (C) Elevated SFRS8 directly downregulated the expression of mouse CACYBP detected by RIP‐qPCR. (D) The expression of mouse CACYBP in RAW264.7 SFRS8‐OE cells compared with Ctrl cells. (E) Chemical structure of methyl3‐{[(4‐methylphenyl) sulfonyl] amino} benzoate (MSAB). (F) TRAP staining demonstrated that MSAB affected osteoclast differentiation in RAW264.7 Ctrl group, RAW264.7+MSAB group, RAW264.7+exosomes group; RAW264.7+exosomes+MSAB group. Scale bar: 500 μM. (G) Statistical analysis on the numbers of osteoclasts. (H) TRAP activity assay examined the differentiation of PMBCs into osteoclasts with the treatment of MSAB upon overexpression of SFRS8. Scale bar: 500 μM. (I) Statistical analysis on the numbers of osteoclasts of PBMCs. (J) qPCR assay was applied to detect CACYBP isoform2 and CACYBP isoform1 expression in PMBCs. Scale bar: 500 μM. The data are expressed as mean ± SD. *p < 0.05, **p < 0.01, ***p < 0.001

To determine the mechanism of SFRS8 modulating osteoclast differentiation, we exploited specific siRNA to attenuate the expression of SFRS8 in RAW264.7 cells. Through mRNA sequencing, Wnt signalling pathway was identified as the potential signalling pathway involved in the regulation of SFRS8 on osteoclast differentiation (Figure 5A). It has been shown that the constitutive activation of β‐catenin in OC promotes the formation of OCs to cause bone loss.^34^ We next evaluated if the regulation of β‐catenin on osteoclast differentiation was related to the isoforms of CACYBP spliced by SFRS8. After compared the mRNA and protein sequences of mouse CACYBP with human CACYBP, we found that the similarity of mouse CACYBP and CACYBP isoform1 reached up to 89% and 93%, respectively, suggesting that mouse CACYBP could be spliced by SFRS8 as well (Figure 5B). The analyses of RNA immunoprecipitation and qPCR corroborated that SFRS8 directly bound to endogenous mouse CACYBP and decreased CACYBP expression in RAW264.7 cells (*p* < 0.05, *p* < 0.01) (Figure 5C,D). As to verify the role of the Wnt signalling pathway in osteoclast differentiation, we used a small molecule inhibitor of Wnt/β‐catenin signal transduction, MSAB,^35^ whose chemical structure was displayed in Figure 5E. Different concentrations of MSAB (0, 2.5, 5, 10, 20, 40 μM) were added to RAW264.7 and ARP1 WT cells. As shown in Figures S5, S6, the expression of β‐catenin was gradually decreased along with the increasing concentrations of MSAB at the protein level. In addition, we tested the effect of MSAB on osteoclast differentiation in RAW264.7 cells treated with MSAB and Exos, alone or both. MSAB impeded RAW264.7 cells differentiating into OCs with or without Exos (*p* < 0.01) (Figure 5F,G).

In agreement of the results in RAW264.7 cells, the increased SFRS8 stimulated human PMBCs to differentiate into OCs in the presence of RANKL and M‐CSF, however the differentiation ability was apparently impaired upon the MSAB treatment examined by the TRAP assay (*p* < 0.01) (Figure 5H,I). Additionally, qPCR assay detected the levels of two isoforms of CACYBP in PBMCs, which demonstrated that CACYBP isoform2 was increased and CACYBP isoform1 was decreased in PBMC SFRS8‐OE cells compared with PBMC control cells (*p* < 0.05) (Figure 5J). Regarding the evidences obtained in RAW264.7 cells and PBMCs, we assume that SFRS8 promotes the alternative splicing of CACYBP to regulate the BM microenvironment through the Wnt/β‐catenin pathway.

### 3.6 Targeted delivery of CACYBP isoform2 siRNA‐loaded exosomes inhibits MM progression

**FIGURE 6:**
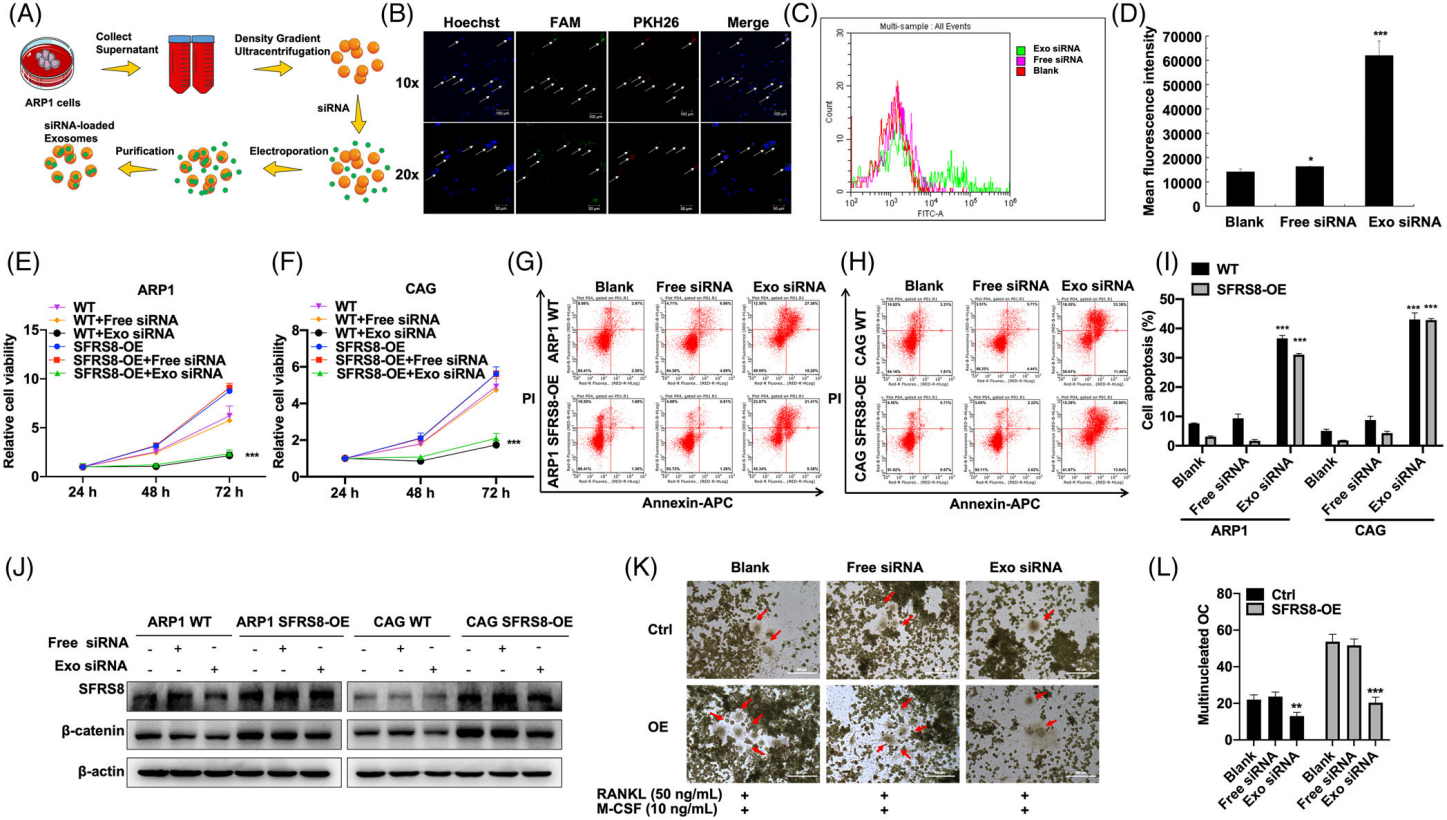
CACYBP isoform2 siRNA‐loaded exosomes inhibit multiple myeloma (MM) proliferation and osteoclast differentiation. (A) Schematic demonstration of the generation of siRNA loaded‐exosomes by electroporation. (B) Intracellular localisation of exosomes by using confocal microscopy. Blue fluorescence: Hoechst staining; green fluorescence: FAM‐siRNA; red fluorescence: PKH26 dye labelled exosomes. Scale bar: 50 μM. (C) Cellular fluorescence for ARP1 cells treated with Free siRNA, Exo siRNA or not recorded by a FACSCalibur flow cytometer. *x*‐axis: cellular fluorescence intensity; *y*‐axis: cell counts. (D) Statistics on fluorescence intensity in different groups. (E,F) MTT assay tested the viability of MM cells treated with Free siRNA, Exo siRNA or not. (G–I) Flow cytometry analysis exhibited the apoptosis of MM cells upon treating with Free siRNA, Exo siRNA or not for 48 h. (J) The expression of β‐catenin protein was examined by WB. (K) TRAP staining revealed that Exo siRNA inhibited osteoclast differentiation. Scale bar: 500 μM. (L) Statistics on the numbers of osteoclasts. The data are shown as mean ± SD. *p < 0.05, **p < 0.01, ***p < 0.001. Exo: exosome

Due to that mature inhibitors targeting splicing factors are seldom available,^36^ we targeted CACYBP isoform2 by using specific siRNA‐loaded Exos to further examine its functions. The detailed steps for loading siRNA into Exos were outlined in Figure 6A. Confocal microscopy was applied to determine the uptake of siRNA‐loaded Exos in ARP1 cells. Extensive internalisation and accumulation of fluorescently labelled FAM‐siRNA were in the cytosol (Figure 6B). The flow cytometry showed that the cells treated with siRNA‐loaded Exos exhibited markedly increased fluorescence intensity compared with the cells treated with free siRNA or not (*p* < 0.001) (Figure 6C,D). Simultaneously, the cellular proliferation was significantly decreased in siRNA‐loaded Exos cells relative to control cells (*p* < 0.001) (Figure 6E,F). In addition, the flow cytometry apoptosis assay proved that targeting CACYBP isoform2 promoted MM cell apoptosis (*p* < 0.001) (Figure 6G–I). Intriguingly, the expression of β‐catenin was decreased in siRNA‐loaded Exos cells compared with the cells treated with free siRNA or not (Figure 6J). The differentiation capacity of RAW264.7 into OCs was alleviated in siRNA‐loaded Exos cells compared with control cells (*p* < 0.01) (Figure 6K,L).

**FIGURE 7:**
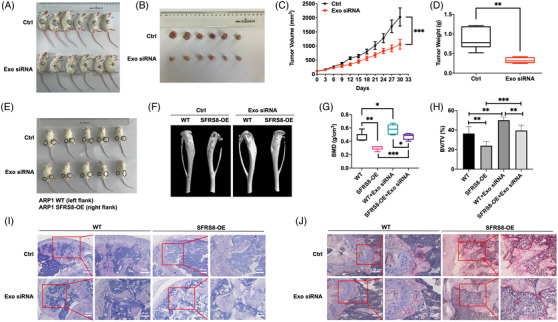
CACYBP isoform2 siRNA‐loaded exosomes inhibit tumor growth in PDX and SCID/NOD‐TIBIA mouse models. (A) Photographic images of PDX model treated by tail i.v. injection with Exo siRNA or not for 30 days. (B) Photographic images of tumours taken from the PDX model in each group. (C) Tumour growth curve of the PDX model in the Ctrl and Exo siRNA groups. (D) Mean tumour weight of the PDX model in the Ctrl and Exo siRNA groups. (E) Schematic images of SCID/NOD‐TIBIA mice in Ctrl and Exo siRNA groups. (F) Representative microCT images of bones in Ctrl and Exo siRNA groups. (G) Bone mineral density (BMD) of SCID/NOD‐TIBIA mice in Ctrl and Exo siRNA groups. (H) Bone volume (BV/TV) of SCID/NOD‐TIBIA mice in the Ctrl and Exo siRNA groups. (I) H&E staining of histological sections of bones in Ctrl and Exo siRNA groups. Scale bar: 100 μM, 50 μM. (J) TRAP staining of histological sections of bones in Ctrl and Exo siRNA groups. Scale bar: 50 μM, 25 μM. The data are shown as mean ± SD. *p < 0.05, **p < 0.01, ***p < 0.001

Subsequently, we evaluated the effect of siRNA‐loaded Exos in vivo by using a PDX model. Patient‐derived primary human MM xenografts were implanted in SCID/NOD mice. CACYBP isoform2 siRNA‐loaded Exos were administered by tail i.v. injection in the PDX model (siRNA 1.5 mg/kg). The tumours in siRNA‐loaded Exos group were much smaller than that in the control group (Figure 7A,B). Consistently, the average volume and weight of tumours in siRNA‐loaded Exos group remarkably lagged behind the tumours in the control group (*p* < 0.001, *p* < 0.01) (Figure 7C,D). In addition, we explored the impact of siRNA‐loaded Exos on the BM microenvironment by using the SCID/NOD‐TIBIA mouse model treated with siRNA‐loaded Exo or not (siRNA 1.5 mg/kg). As Figure 7E shown, there were no significant changes of swelling and foot valgus between Exo siRNA group and control group. However, the analysis of μCT showed that siRNA‐loaded Exos reduced bone erosion to a large extent (Figure 7F). The BMD and BV/TV of mice were significantly increased upon injection of siRNA‐loaded Exo (*p* < 0.05, *p* < 0.01, *p* < 0.001) (Figure 7G,H). The results of H&E and TRAP staining showed the low density of tumour cells (Figure 7I) and the diminished multinucleated OCs in the siRNA‐loaded Exos group (Figure 7J) compared with the control group, respectively. Summarily, these results suggest that CACYBP siRNA‐loaded Exos may suppress MM cell growth and improve the BM microenvironment in vivo.

## 4 Discussion

MM remains an incurable hematological malignancy featured by the malignant proliferation of plasma cells and strong dependence on BM.^37^ Gene expression profiles have provided novel insight into the tumour biology and potential druggable targets in MM,^38^ however, only 63 critical drivers are recently identified.^39^ In a subset of specimens, the undetected drivers may be located in the non‐coding genome or include an ambiguous mechanism.^40^ In general, alternative splicing contributes to cell differentiation, however abnormal alternative splicing is closely related to the occurrence of tumours and the destruction of the protein interaction pathway during the tumour development.^41^, ^42^ Abnormal alternative splicing events may produce multiple abnormal proteins, and these proteins are regarded as diagnostic markers and novel therapeutic targets in various cancers.^43^, ^44^ Targeted splicing is a kind of the RNA‐based therapeutic approach, usually including shRNA interference, siRNA,^45^ clustered regularly interspaced short palindromic repeats (CRISPR)‐Cas directed gene editing,^46^ and even single‐base editors (BEs) cytosine‐BEs (CBEs) or adenine‐BEs (ABEs),^47^, ^48^ as well as splice‐switching oligonucleotides^49^, ^50^ and small molecules^51^ that modulate splicing. However, off‐target effects and unefficient delivery to the target organs still remain as the major challenges.^52^

SFRS8 is a splicing factor regulating mRNA splicing.^9^, ^13^ In the present study, we found that SFRS8 was abnormally increased in clinical MM samples and its elevation was significantly associated with poor prognosis of MM patients. Moreover, we combined the analyses of RIP‐seq and MM GEP cohorts to screen out CACYBP as the key target of SFRS8. CACYBP is widely involved in the regulation of cell proliferation, differentiation, skeletal rearrangement, substrate ubiquitination modification and other cell processes by binding different protein substrates.^53^ We validated the existence of two CACYBP splicing isoforms, CACYBP isoform1 (NM_014412.3) and CACYBP isoform2 (NM_001007214.1). The distinct protein isoforms yielded by the identical gene through alternative splicing may possess related, different or even opposite functions.^54^ The conserved domains in both isoforms of CACYBP guarantee the function of promoting ubiquitination. However, the ubiquitination function of CACYBP isoform2 is much weaker than CACYBP isoform1. We found that SFRS8 promoted MM cell proliferation via CACYBP isoform2, and CACYBP isoform1 triggered the anti‐MM activity in a different way. Increased SFRS8 directly bound to CACYBP isoform2 and promoted its expression, in contrast the effect of CACYBP isoform1 was weak due to competitive inhibition. Therefore, it was mainly executed by elevated CACYBP isoform2 leading to a decrease in the ubiquitination and degradation of β‐catenin.

The progression of MM largely relies on the BM microenvironment,^55^ which communicates through different factors including extracellular vesicles to support the growth of MM cells.^56^ Therefore, it is of great importance to explore novel therapeutic strategies that not only treat osteolysis, but also alleviate tumour growth by affecting the BM microenvironment. Overwhelming evidences demonstrate that molecular interactions between MM cells and the BM microenvironment induce MM pathological changes and related complications exemplified by bone lesions due to osteolysis.^57^, ^58^ The BM contains mesenchymal stromal cells (MSC), osteoblasts (OBs), OCs, endothelial cells and immune system cells, which support the differentiation, migration, proliferation, survival and drug resistance of MM cells.^59^ Exos are termed as a crucial component of the BM microenvironment, which differ prominently between MM patients and healthy individuals.^60^ Many Exos have been identified in the peripheral blood and BM of MM patients, which promote cell proliferation, angiogenesis and immunosuppression, and maintain a favourable BM microenvironment for the development of MM.^30^, ^61^ Exos derived from myeloma cells have been seen to cause osteolysis and enhance the activity of OCs in MM patients.^62^ Recently, Exos are used as new drugs and siRNA delivery vehicles to target cancer cells.^63^, ^64^ The striking finding of our study was that SFRS8 was secreted by MM cells through Exos to promote osteoclast differentiation, thereby favouring MM cell proliferation.

The BM stromal cells secrete Wnt ligands to activate Wnt signalling in MM, which is mediated by the transcriptional effector β‐catenin.^65^ Dysregulation of Wnt/β‐catenin signalling presents in several types hematological malignancies including MM,^66^, ^67^ which promotes MM cell proliferation^68^ and exerts a direct effect on the formation of OCs and pre‐osteoclast proliferation.^69^ Altering β‐catenin stabilisation in OCs may promote osteoclastogenesis.^34^ In this study, we found that SFRS8 promoted the differentiation of OCs via inducing CACYBP isoform2 to increase β‐catenin. In particular, the Wnt/β‐catenin pathway was also confirmed to be involved in the regulation of SFRS8‐Exos on osteoclast differentiation.

A key opportunity for the future will be to discover the novel inhibitors of RNA splicing factors including SFRS8 for the preclinical and clinical applications. The application of siRNAs with gene‐silencing has opened a new insight in drug discovery, but it is obstructed by its instability, inaccurate tissue‐specific delivery and the possibility of inducing an immune response.^70^ As Exos can naturally carry RNA between cells with good stability in blood, they are regarded as promising tools for delivery of therapeutic siRNA to the target cells. Compared with the commonly used artificial liposomes and nanoparticles for siRNA delivery, Exos allow more effective delivery after systemic administration.^71^ We observed that the application of siRNA‐loaded Exos to target alternative splicing achieved well curative effect in osteolysis in the clinically relevant animal models. Exo‐mediated RNA delivery will be required as a desirable anti‐MM therapy, especially for targeted therapy of alternative splicing in the BM microenvironment.

## 5 Conclusions

**FIGURE 8:**
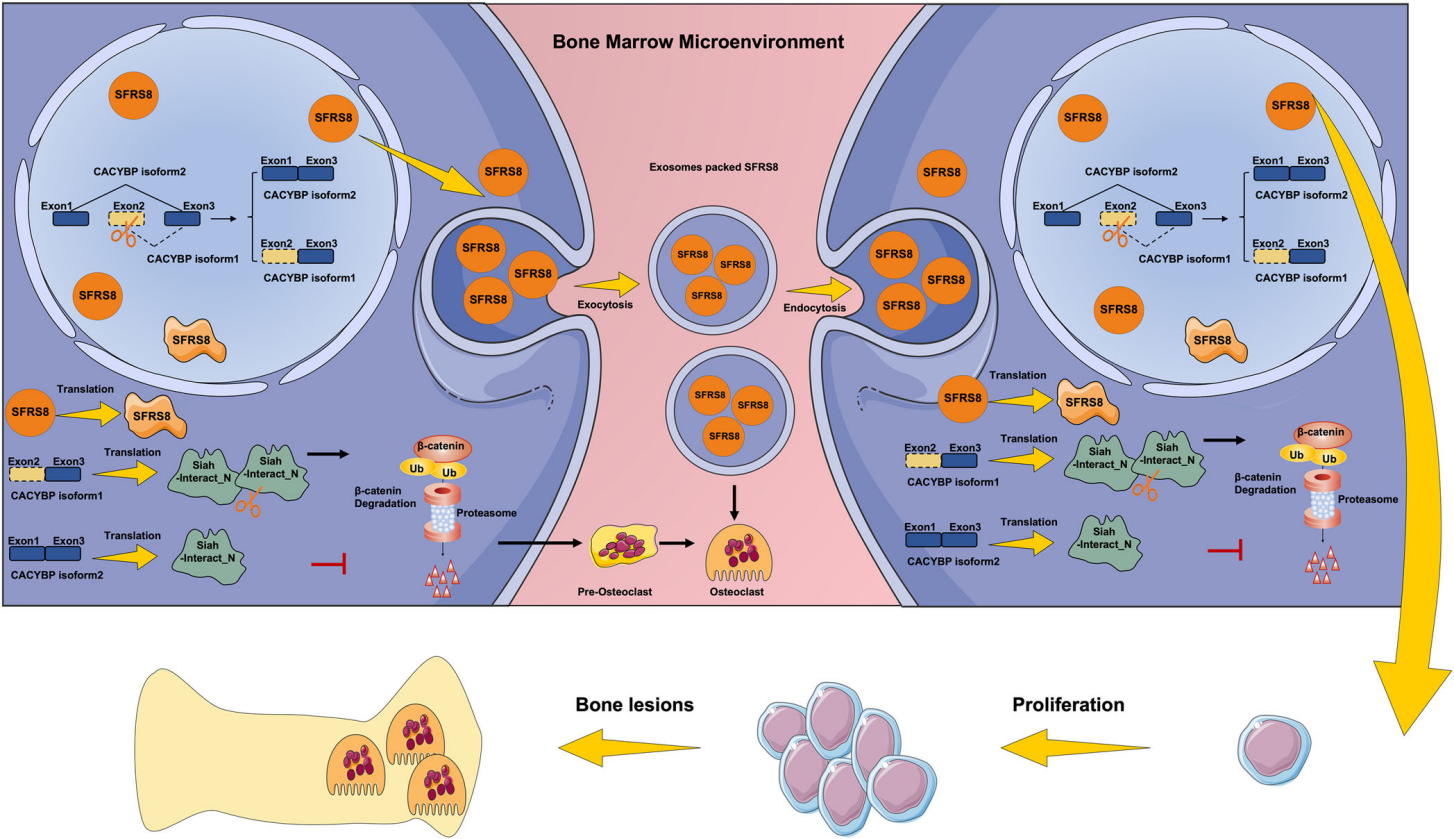
Schematic depiction illustrates that splicing factor arginine/serine‐rich 8 (SFRS8) is a promising therapeutic target for bone marrow (BM) microenvironment in multiple myeloma (MM) via regulating CACYBP exon skipping to inhibit β‐catenin ubiquitination and promoting osteoclast differentiation through exosomes

In summary, SFRS8 plays a role in facilitating the progression of MM cells and osteoclast differentiation via regulating alternative splicing of CACYBP. Our findings demonstrate that exosomal siRNA of CACYBP isoform2 effectively affects MM cell proliferation, bone lesion formation and pathologic changes in the BM microenvironment. Certainly, targeting the SFRS8/CACYBP/β‐catenin axis during the MM development may be a promising strategy to improve MM patient outcomes (Figure 8).

## CONFLICT OF INTERESTS

The authors declare that there is no conflict of interest that could be perceived as prejudicing the impartiality of the research reported.

## Supporting information



Supporting Information

Supporting Information

Supporting Information

Supporting Information

Supporting Information

Supporting Information

Supporting Information

Supporting Information
