# Correction to“Splicing factor arginine/serine‐rich 8 promotes multiple myeloma malignancy and bone lesion through alternative splicing of CACYBP and exosome‐based cellular communication”

**DOI:** 10.1002/ctm2.1282

**Published:** 2023-05-25

Yuanjiao Zhang, Xichao Yu, Rongze Sun, Jie Min, Xiaozhu Tang, Zigen Lin, Siyuan Xie, Xinying Li, Shengfeng Lu, Zhidan Tian, Chunyan Gu, Lesheng Teng, Ye Yang. Splicing factor arginine/serine‐rich 8 promotes multiple myeloma malignancy and bone lesion through alternative splicing of CACYBP and exosome‐based cellular communication. Clin Transl Med. 2022;12(2):e684. https://doi.org/10.1002/ctm2.684

Following publication of the original article ^1^, the authors identified minor errors in the figures, specifically:

- Figure 1C: one incorrect image was misemployed. Please check the Supplementary file 1 for the instruction and original data.
- Supporting information Figure S3 (upper panel of Figure S3): two incorrect images were misemployed. Please check the Supplementary file 2 for the instruction and original data.

The corrected figures are given below. The authors provided the Journal with the original data files. The corrected figures are provided here. The correction does not have any effect on the results or conclusions of the paper. The original article has been corrected.

We apologize for these errors.

**FIGURE 1:**
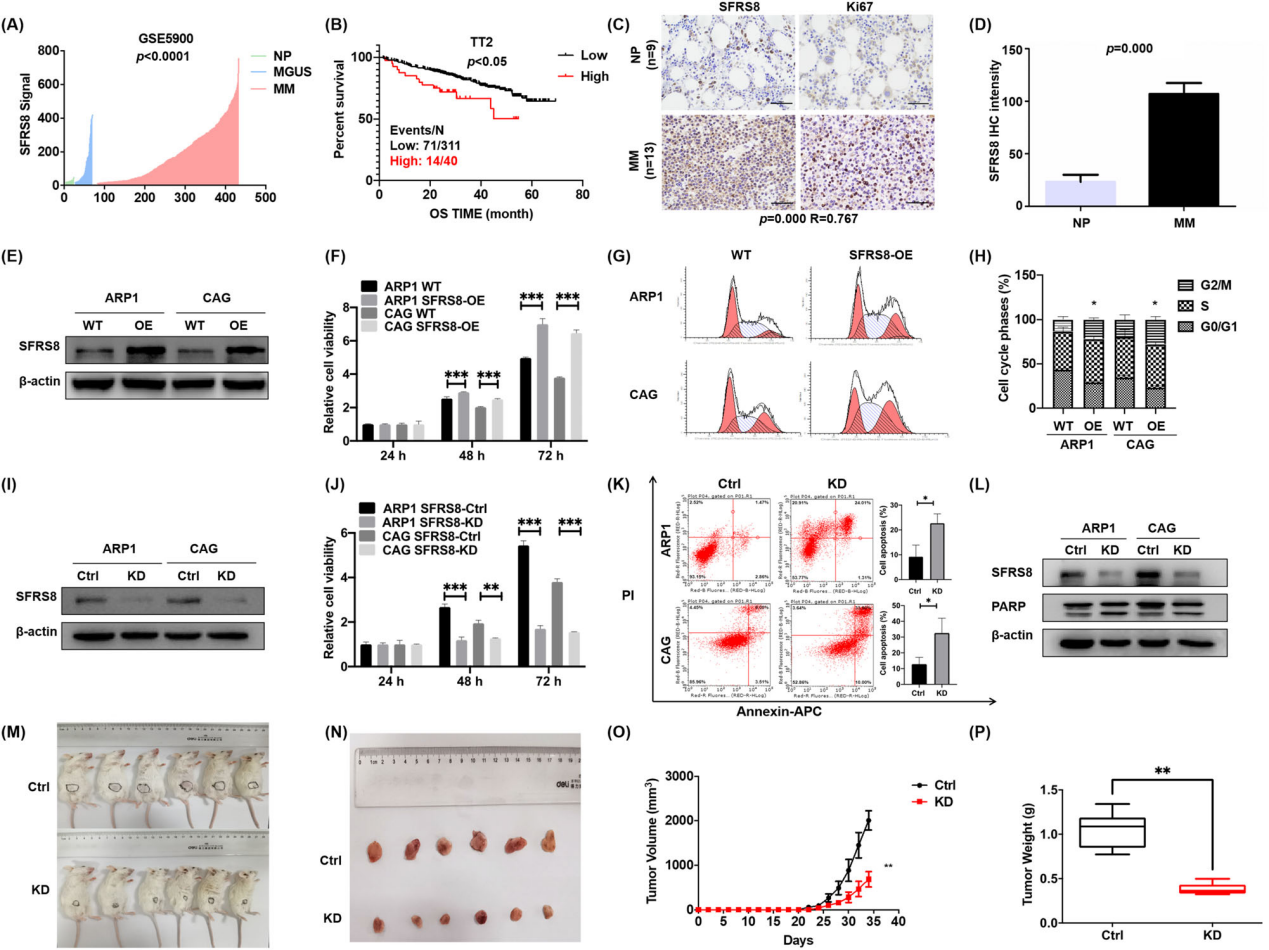
.

## Supporting information

Supporting Information

Supporting Information

Supporting Information
