# Supplementary material for: Splicing factor arginine/serine‐rich 8 promotes multiple myeloma malignancy and bone lesion through alternative splicing of CACYBP and exosome‐based cellular communication
Source: Clin Transl Med. 2022 Feb 20;12(2):e684. doi: 10.1002/ctm2.684 (PMC8858635; doi:10.1002/ctm2.684)
Supplement: Supplementary file 1 — Supporting Information [file CTM2-12-e684-s002.docx]

**Supplementary Materials and Methods**

**Preparation of siRNA-loaded Exosomes**

A Neon electroporation system was utilized in this study. The exosomes were mixed with the Neon electroporation buffer at a 1:1 ratio. FAM labeled siRNA was added to the mixture at a final concentration of FAM-siRNA: Exosomes=200 nM: 20 μg/mL. Then electroporation was performed at 100 V, 50 uF with the pulse width set at 30 ms for three times including two-second pause. Following electroporation, one unit of RNase A was added to the mixture to eliminate free siRNA outside the exosomes. EDTA was added to reduce the undesirable electroporation-induced siRNA precipitation during the loading process.^1,2^

**Electrotransmitter method**

BTXpress Cytoporation Media T4 (BTX, 47-0003) was used to resuspend cells in 1×10^6^/mL, added siRNA to a final concentration of 100 nM, and transferred to electric shock cup after mixed and transfected finally. Electric transfer parameters were determined as following: square wave, voltage 960 volts; duration, 0.1 ms; numbers of pulse, 2; pulse interval, 1.0 sec; electrode gap, 4mm.

**Western blotting (WB)**

The total protein was extracted and quantified by Microvolume Spectrometer (Berthold, Germany). 20 μg protein was resolved by SDS-PAGE and transferred onto a 0.45 μm PVDF membrane (Millipore, Bedford, MA). After the membrane was blocked with 5% non-fat milk for 1 h, it was incubated with primary antibodies overnight and then with secondary antibodies for 1 h. Enhanced Chemiluminescence (ECL) Detection Kit (Amersham Pharmacia Biotech, Piscataway, NJ) was used to detect the bands.

**Hematoxylin and eosin (H&E) staining**

H&E staining steps were as follows: the tissues were undergone decalcification and dissection before treated by paraffin; then the tissues were sectioned into pieces with 5‐10 μm thickness; after xylene‐deparaffinizing at 37°C for 20 min, the sections were stained using H&E Staining Kit (Sangon Biotech, Shanghai, China). Inverted fluorescence microscope (Optika IM-3FL4, Thermo, USA) was applied for morphological observation.

**Transcriptomic RNA Sequencing**

we exploited specific siRNA to attenuate the expression of SFRS8 in RAW264.7 cells and then performed mRNA sequencing. mRNA was extracted from total RNA following the removal of rRNA. After constructing an RNA-sequencing library, the Illumina NovaSeq 6000 platform was utilized for PE150 sequencing. Data were analyzed using strict data quality controls to identify several differentially expressed genes (DEGs). All data analysis and processing were performed by lc-bio (Hangzhou, China).

**KEGG pathway enrichment analysis**

Kyoto Encyclopedia of Genes and Genomes (KEGG), It is a public database on genome deciphering. The website is [www.genome.jp/kegg](http://www.genome.jp/kegg). KEGG is the main public database about Pathway. Pathway significant enrichment analysis takes KEGG Pathway as the unit and applies hypergeometric test to find pathways that are significantly enriched in significantly differently expressed genes compared with the entire genome background.

**GO function significance enrichment analysis**

Gene Ontology (GO) is an internationally standardized gene function classification system, which provides a set of dynamically updated controlled vocabulary to comprehensively describe the attributes of genes and gene products in organisms. Gene Ontology (GO) sets Biological Process (BP), Cellular Component (CC) and Molecular Function (MF) were used for the analysis. The basic unit of GO is term, and each term corresponds to an attribute. GO functional significance enrichment analysis firstly maps all significantly differentially expressed genes to each term in the Gene Ontology database, calculates the number of genes in each term, and then applies hypergeometric test to find out the significance compared with the background of the entire genome. GO entries that are significantly enriched in differentially expressed genes.

**References**

1. Yang Z, Xie J, Zhu J, et al. Functional exosome-mimic for delivery of siRNA to cancer: in vitro and in vivo evaluation. *J Control Release*. Dec 10 2016;243:160-171. doi:10.1016/j.jconrel.2016.10.008

2. Kooijmans SAA, Stremersch S, Braeckmans K, et al. Electroporation-induced siRNA precipitation obscures the efficiency of siRNA loading into extracellular vesicles. *J Control Release*. Nov 28 2013;172(1):229-238. doi:10.1016/j.jconrel.2013.08.014
