# Supplementary material for: Splicing factor arginine/serine‐rich 8 promotes multiple myeloma malignancy and bone lesion through alternative splicing of CACYBP and exosome‐based cellular communication
Source: Clin Transl Med. 2022 Feb 20;12(2):e684. doi: 10.1002/ctm2.684 (PMC8858635; doi:10.1002/ctm2.684)
Supplement: Supplementary file 2 — Supporting Information [file CTM2-12-e684-s001.docx]

**Supplementary Tables**

**Supplementary Table S1**

Table S1 Sequences of the primers used in the study

| Targets | Sequences |
| --- | --- |
| CACYBP isoform2 (NM_001007214.1)-F | CCTCCACGCTTAACATATC |
| CACYBP isoform2 (NM_001007214.1)-R | GCAACACCTTTACCTCTTC |
| CACYBP isoform1 (NM_014412.3)-F | GAAGGTTCGAGATCCGTC |
| CACYBP isoform1 (NM_014412.3)-R | GGGCATCACGTACTCTTT |
| GAPDH-F | GGGGAGCCAAAAGGGTCATCATCT |
| GAPDH-R | GACGCCTGCTTCACCACCTTCTTG |
| CACYBP(MOUSE)-F | CTTGCGAAGGTTCCAGAG |
| CACYBP(MOUSE)-R | CTTGTAAGAGTATCACGTAGTCT |
| GAPDH(MOUSE)-F | AACCTGCCAAGTATGATGA |
| GAPDH(MOUSE)-R | GGAGTTGCTGTTGAAGTC |
| SFRS8-F | CGTAATAGTCGCTACCGTAAGTGA |
| SFRS8-R | AGGCAAGGCAAGAGGAAGAA |

**Supplementary Table S2**

Table S2 The sequences of siRNAs used in the study

| Targets | Sequences |
| --- | --- |
| CACYBP isoform2 (NM_001007214.1)  siRNA | sense (5'-3'):  CUCUAGUCAACUUCCGACUTT |
|  | antisense (5'-3'):  AGUCGGAAGUUGACUAGAGTT |
| SFRS8(MOUSE) siRNA | sense (5'-3'):  GCCAACCUCUGACAACGAATT |
|  | antisense (5'-3'):  UUCGUUGUCAGAGGUUGGCTT |
| Negative control | sense (5'-3'):  UUCUCCGAACGUGUCACGUTT |
|  | antisense (5'-3'):  ACGUGACACGUUCGGAGAATT |

**Supplementary Table S3**

**Patient characteristics**

MM patient was male, 67 years old, multiple myeloma IgA-κ type DS stage III stage B group ISS stage III. The clinical and hematological characteristics are shown in Table S3.

Table S3 Clinical and hematological characteristics of the patient.

| Sex/age | Male/67 |
| --- | --- |
| Subtype | IgA, κ |
| Immunoglobulin, IgA, g/L | 37.70 |
| β2- microglobulin, mg/L | 13.40 |
| Urine κ light chain, mg | 1780 (24h urine volume 2265ml) |
| Urine protein, mg/L | 1034.40 |
| Urine creatinine, μmol/L | 2843.5 |
| Creatinine clearance rate, mL/min | 12.56 |
| Promyelocytic plasma cells, % | 54 |
| Abnormal plasma cells, % | 7.9 |

**Supplementary Table S4**

**Gene chip data**

The Probe 201382_at was obtained from Affymetrix's gene chip, chip platform [HG-U133_Plus_2] Affymetrix Human Genome U133 Plus 2.0 Array.

Table S4 The information of probe 201382_at

| ID | 201382_at |
| --- | --- |
| GB_ACC | NM_014412 |
| SPOT_ID |  |
| Species Scientific Name | Homo sapiens |
| Annotation Date | 6-Oct-14 |
| Sequence Type | Exemplar sequence |
| Sequence Source | GenBank |
| Target Description | gb: NM_014412.1 /DB_XREF=gi:7656951 /GEN=CACYBP /FEA=FLmRNA /CNT=247 /TID=Hs.27258.0 /TIER=FL /STK=1 /UG=Hs.27258 /LL=27101 /DEF=Homo sapiens calcyclin binding protein (CACYBP), mRNA. /PROD=calcyclin binding protein /FL=gb:AF057356.1 gb: NM_014412.1 gb:AF314752.1 |
| Representative Public ID | NM_014412 |
| Gene Title | calcyclin binding protein |
| Gene Symbol | CACYBP |
| ENTREZ_GENE_ID | 27101 |
| RefSeq Transcript ID | NM_001007214 /// NM_014412 /// XM_005245092 |
| Gene Ontology Biological Process | 0007507 // heart development // inferred from electronic annotation /// 0007568 // aging // inferred from electronic annotation /// 0045740 // positive regulation of DNA replication // inferred from electronic annotation /// 0055007 // cardiac muscle cell differentiation // inferred from electronic annotation /// 0060416 // response to growth hormone // inferred from electronic annotation /// 0060548 // negative regulation of cell death // inferred from electronic annotation /// 0071277 // cellular response to calcium ion // inferred from electronic annotation |
| Gene Ontology Cellular Component | 0005634 // nucleus // inferred from direct assay /// 0005641 // nuclear envelope lumen // inferred from electronic annotation /// 0005730 // nucleolus // inferred from direct assay /// 0005737 // cytoplasm // inferred from direct assay /// 0030877 // beta-catenin destruction complex // inferred from direct assay /// 0043005 // neuron projection // inferred from electronic annotation /// 0044297 // cell body // inferred from electronic annotation /// 0070062 // extracellular vesicular exosome // inferred from direct assay |
| Gene Ontology Molecular Function | 0005515 // protein binding // inferred from physical interaction /// 0042803 // protein homodimerization activity // inferred from physical interaction |
