# Supplementary figures and images for: Splicing factor arginine/serine‐rich 8 promotes multiple myeloma malignancy and bone lesion through alternative splicing of CACYBP and exosome‐based cellular communication
Source: Clin Transl Med. 2022 Feb 20;12(2):e684. doi: 10.1002/ctm2.684 (PMC8858635; doi:10.1002/ctm2.684)

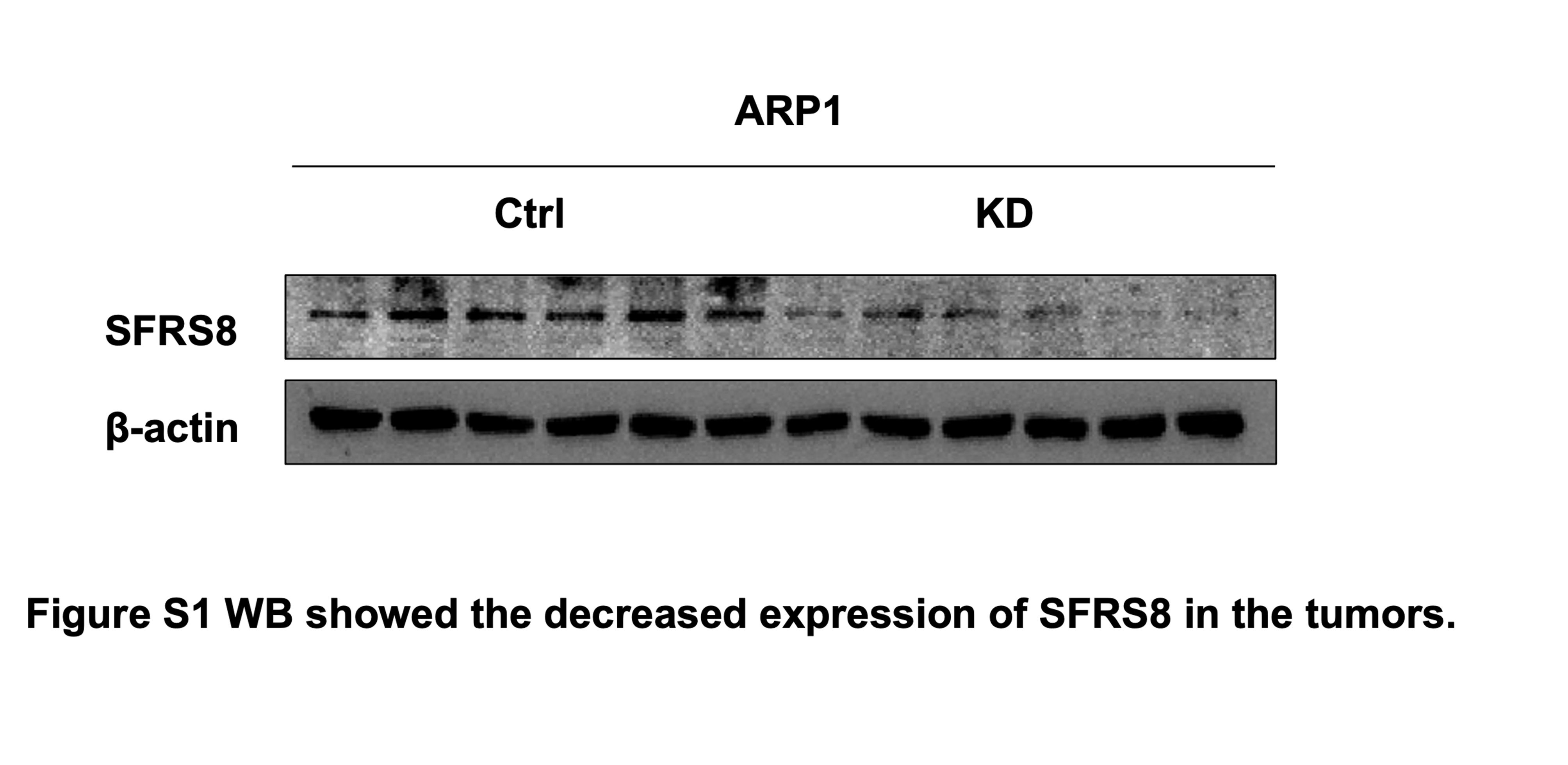

Supplement: Supplementary file 3 — Supporting Information [file CTM2-12-e684-s008.tif]

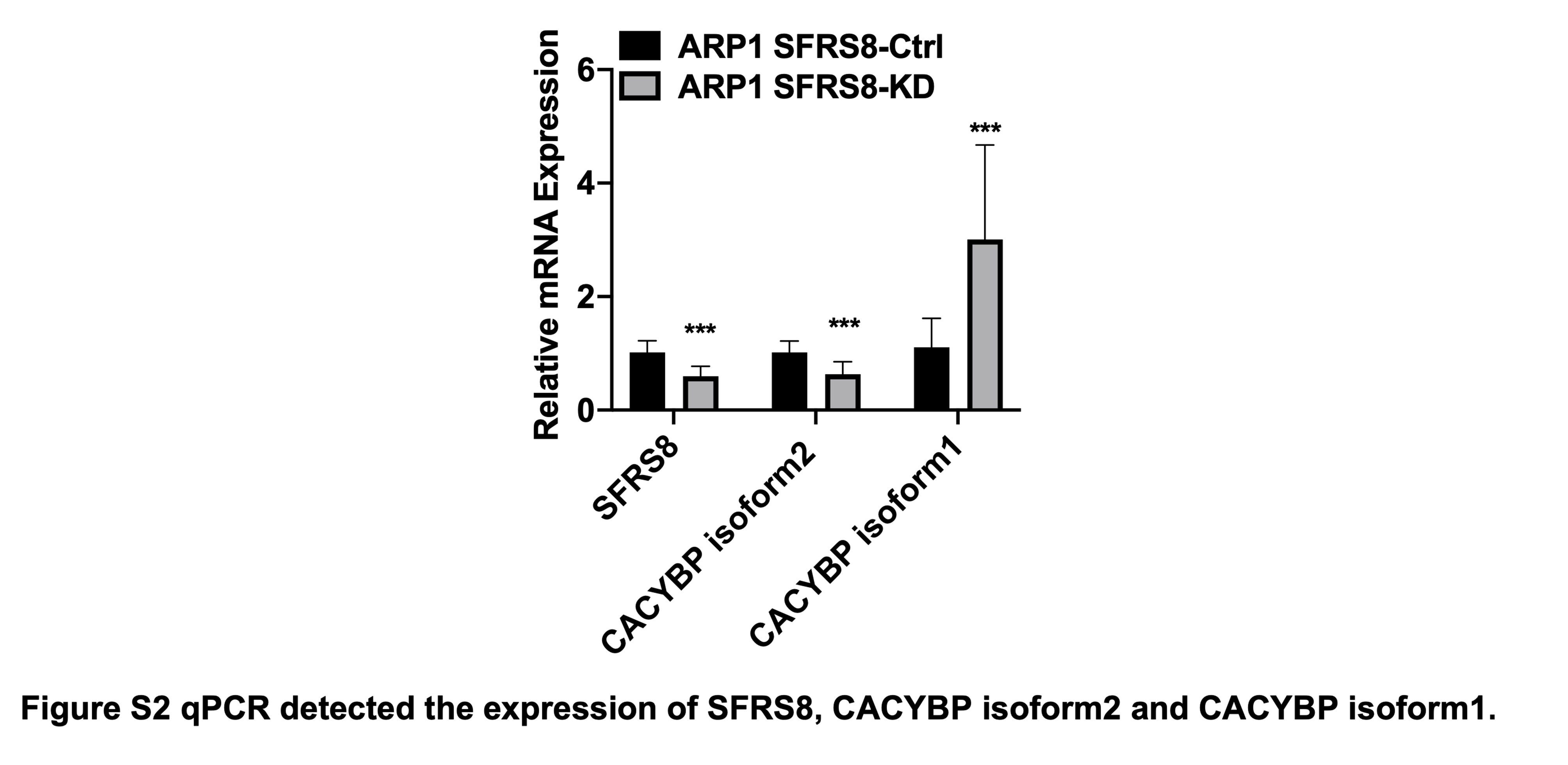

Supplement: Supplementary file 4 — Supporting Information [file CTM2-12-e684-s007.tif]

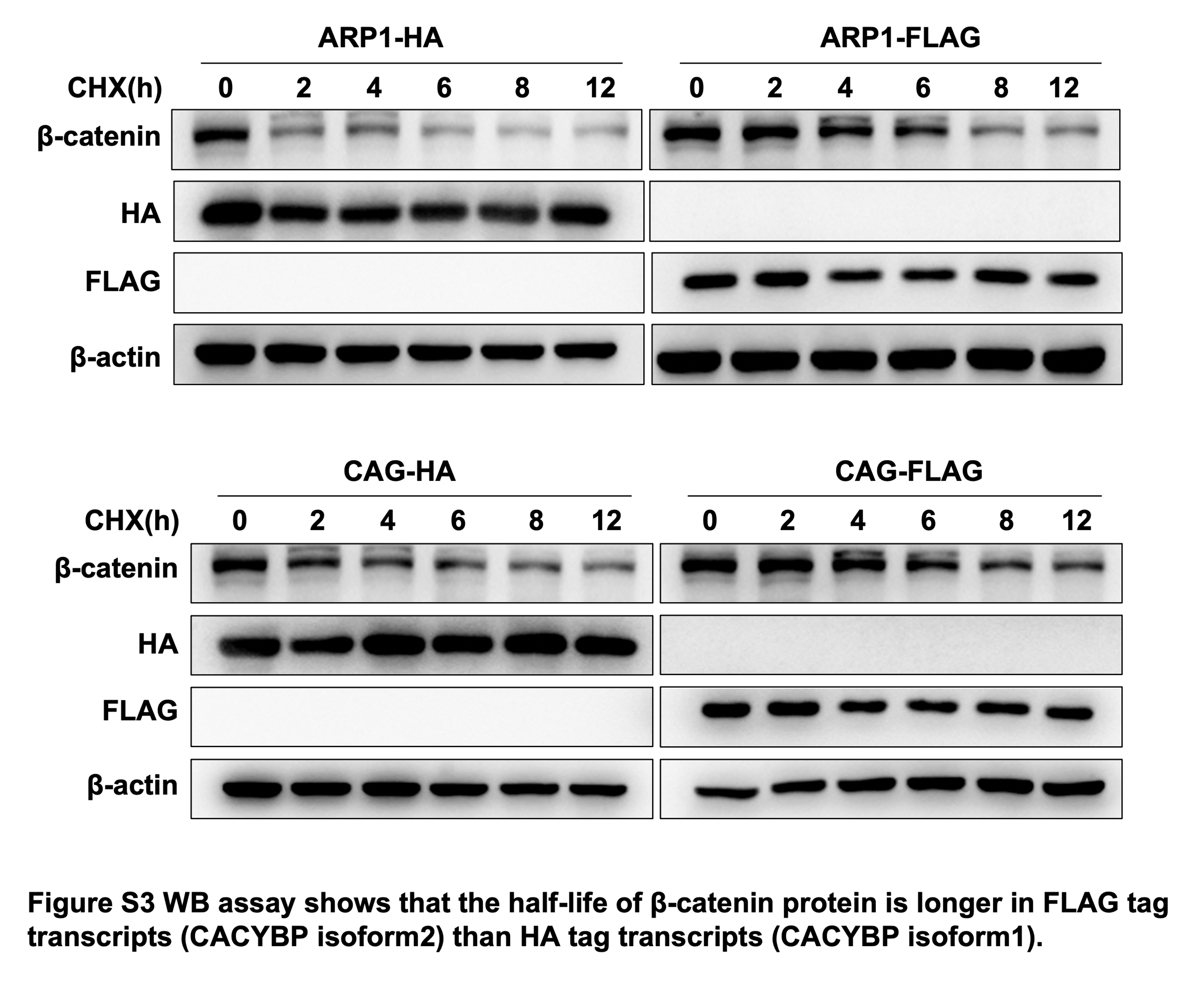

Supplement: Supplementary file 5 — Supporting Information [file CTM2-12-e684-s006.tif]

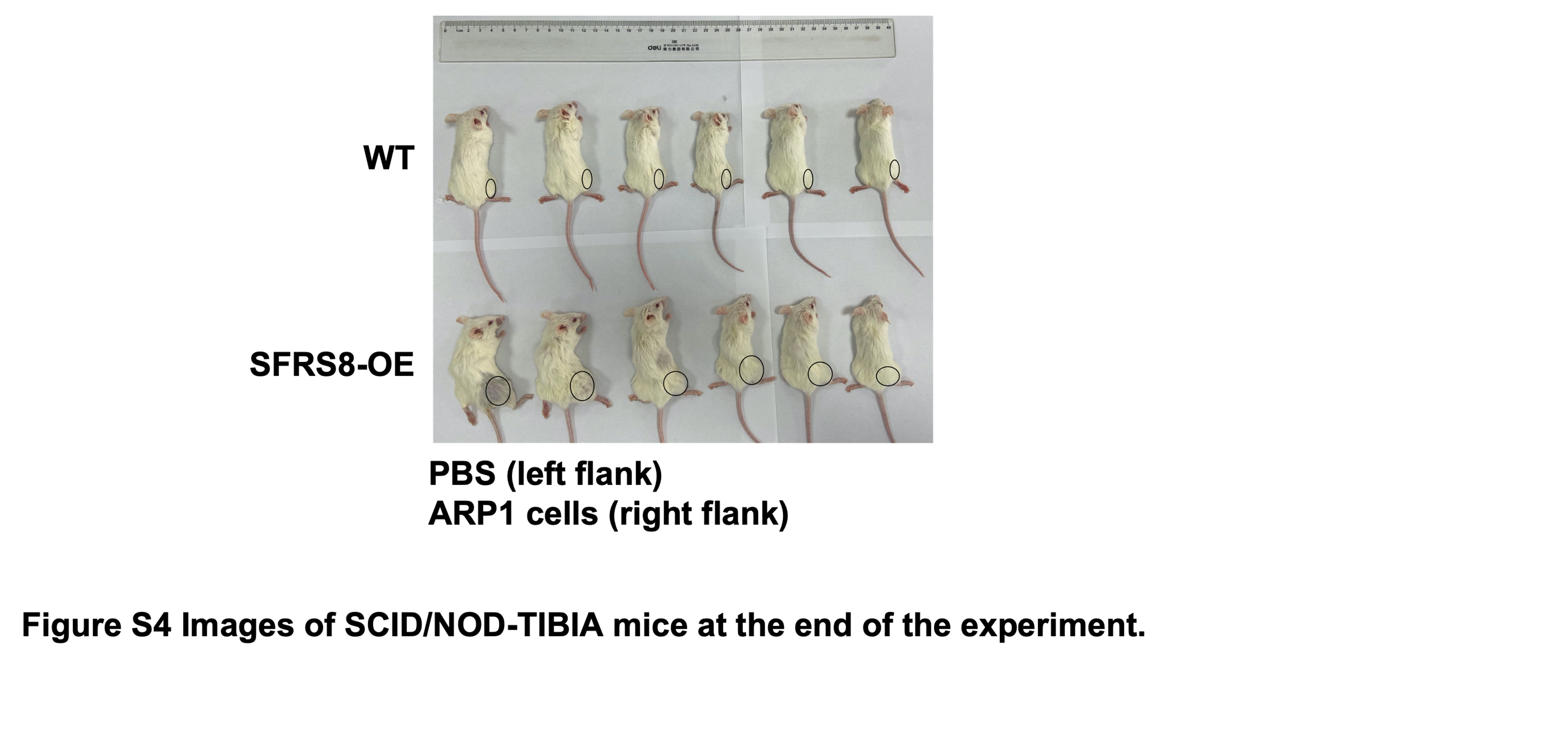

Supplement: Supplementary file 6 — Supporting Information [file CTM2-12-e684-s003.tif]

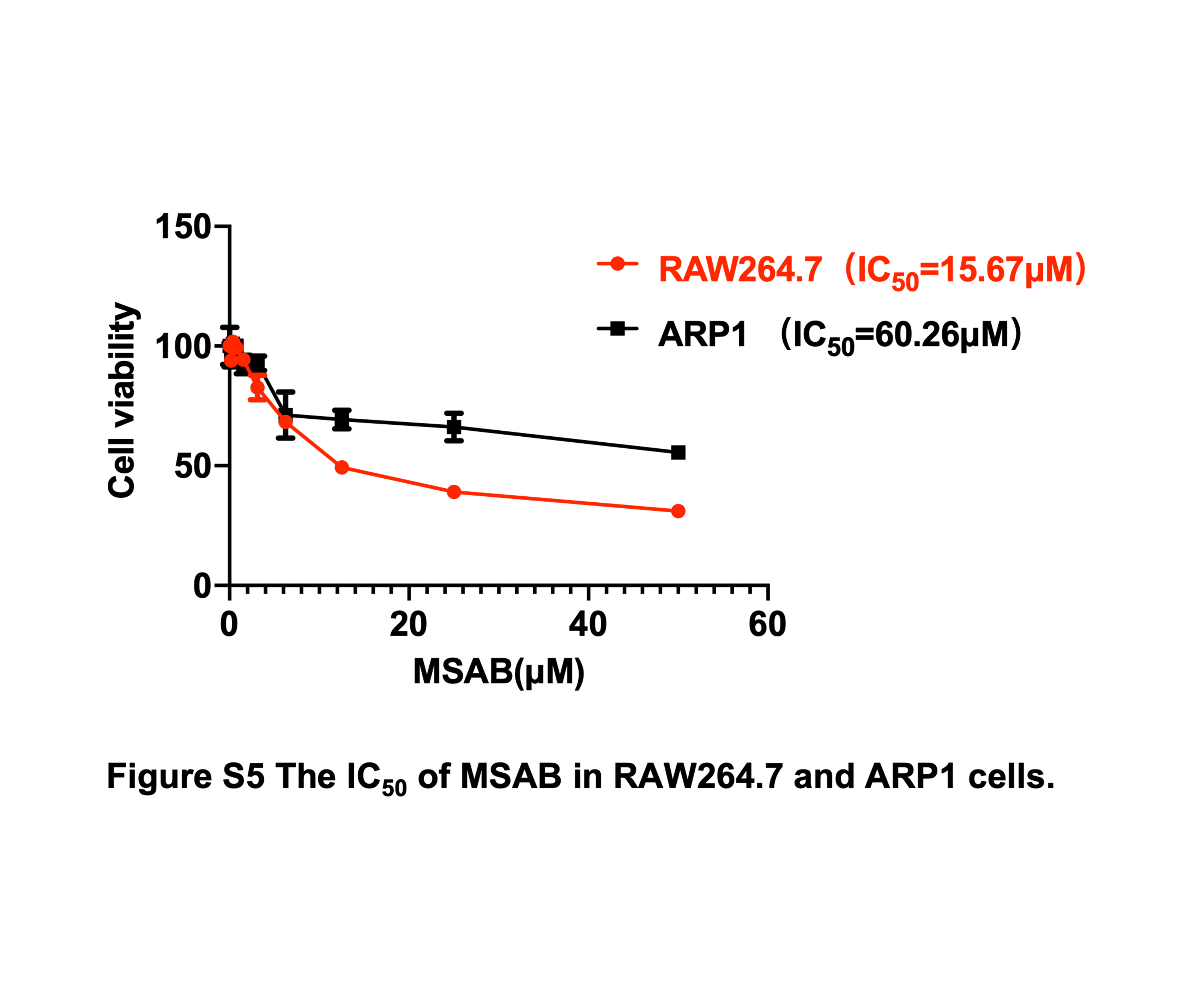

Supplement: Supplementary file 7 — Supporting Information [file CTM2-12-e684-s004.tif]

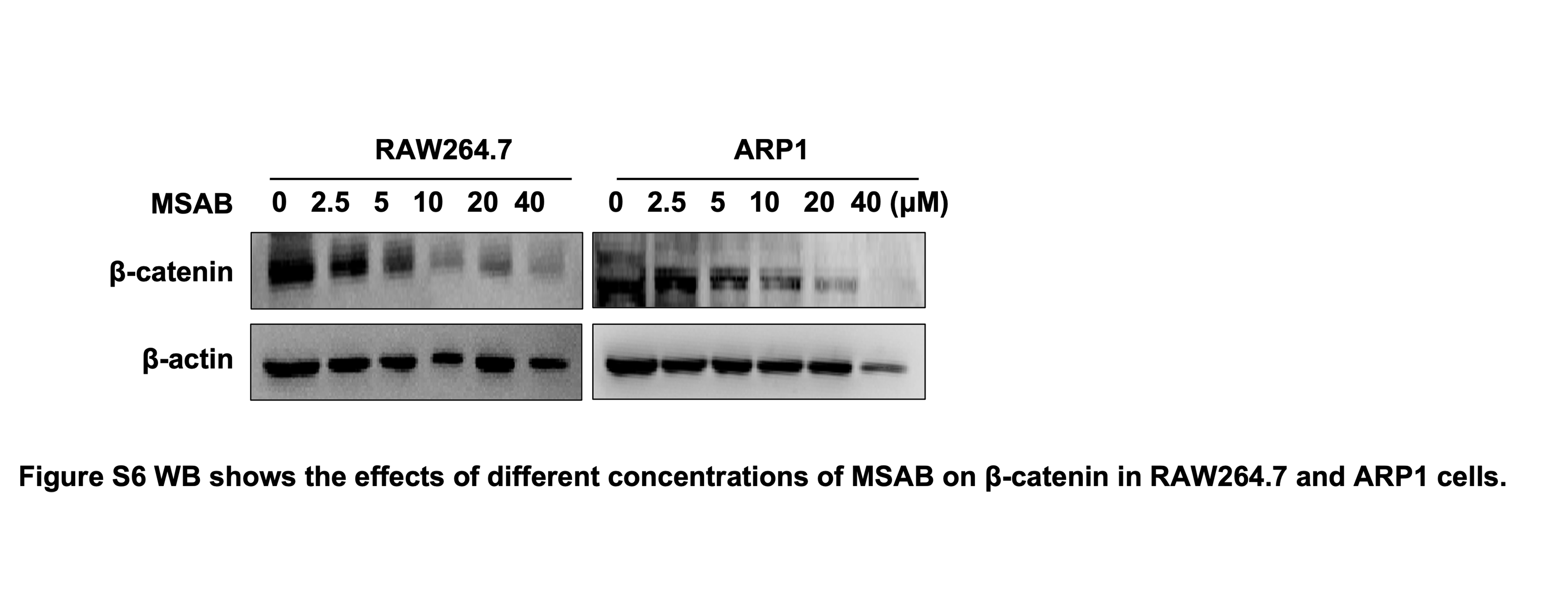

Supplement: Supplementary file 8 — Supporting Information [file CTM2-12-e684-s005.tif]
