# Supplementary figures and images for: Correction to“Splicing factor arginine/serine‐rich 8 promotes multiple myeloma malignancy and bone lesion through alternative splicing of CACYBP and exosome‐based cellular communication”
Source: Clin Transl Med. 2023 May 25;13(5):e1282. doi: 10.1002/ctm2.1282 (PMC10212051; doi:10.1002/ctm2.1282)

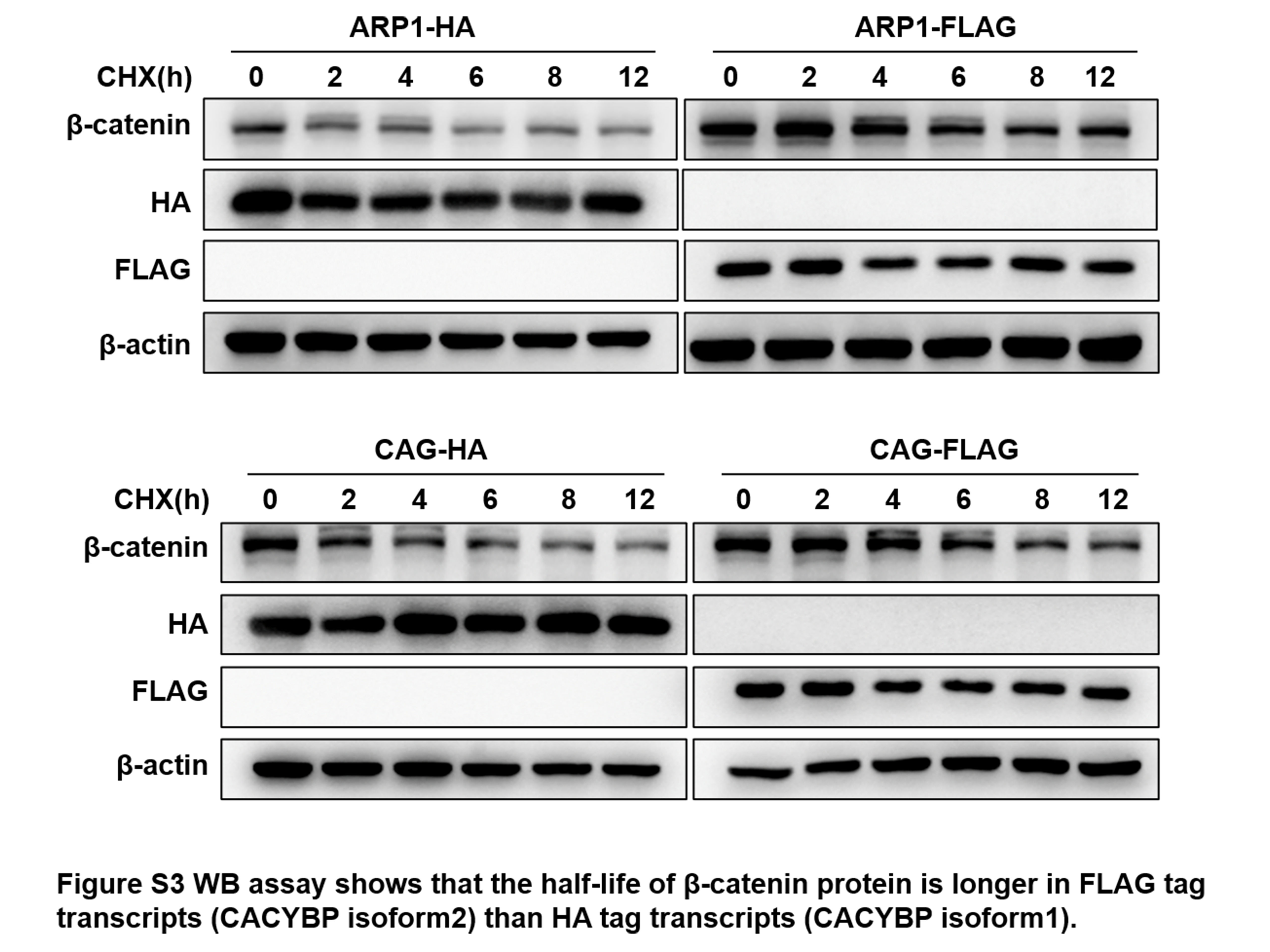

Supplement: Supplementary file 1 — Supporting Information [file CTM2-13-e1282-s003.tif]
