# Supplementary material for: Correction to“Splicing factor arginine/serine‐rich 8 promotes multiple myeloma malignancy and bone lesion through alternative splicing of CACYBP and exosome‐based cellular communication”
Source: Clin Transl Med. 2023 May 25;13(5):e1282. doi: 10.1002/ctm2.1282 (PMC10212051; doi:10.1002/ctm2.1282)
Supplement: Supplementary file 2 — Supporting Information [file CTM2-13-e1282-s002.docx]

**Supplementary file 1 for the instruction and original data of Figure 1C**

We identified one error in Figure 1C as following:

Two pictures of Ki67 (NP) and Ki67 (MM) (1^st^ row, 2^nd^ column, 2^nd^ row, 2^nd^ column) were misemployed.

**
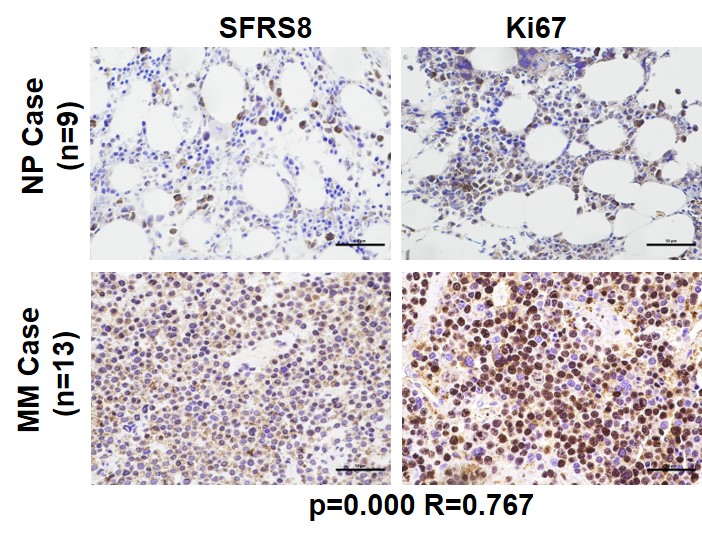
**

**Figure 1C in the published manuscript**

We checked the original records and collected the data as follows.


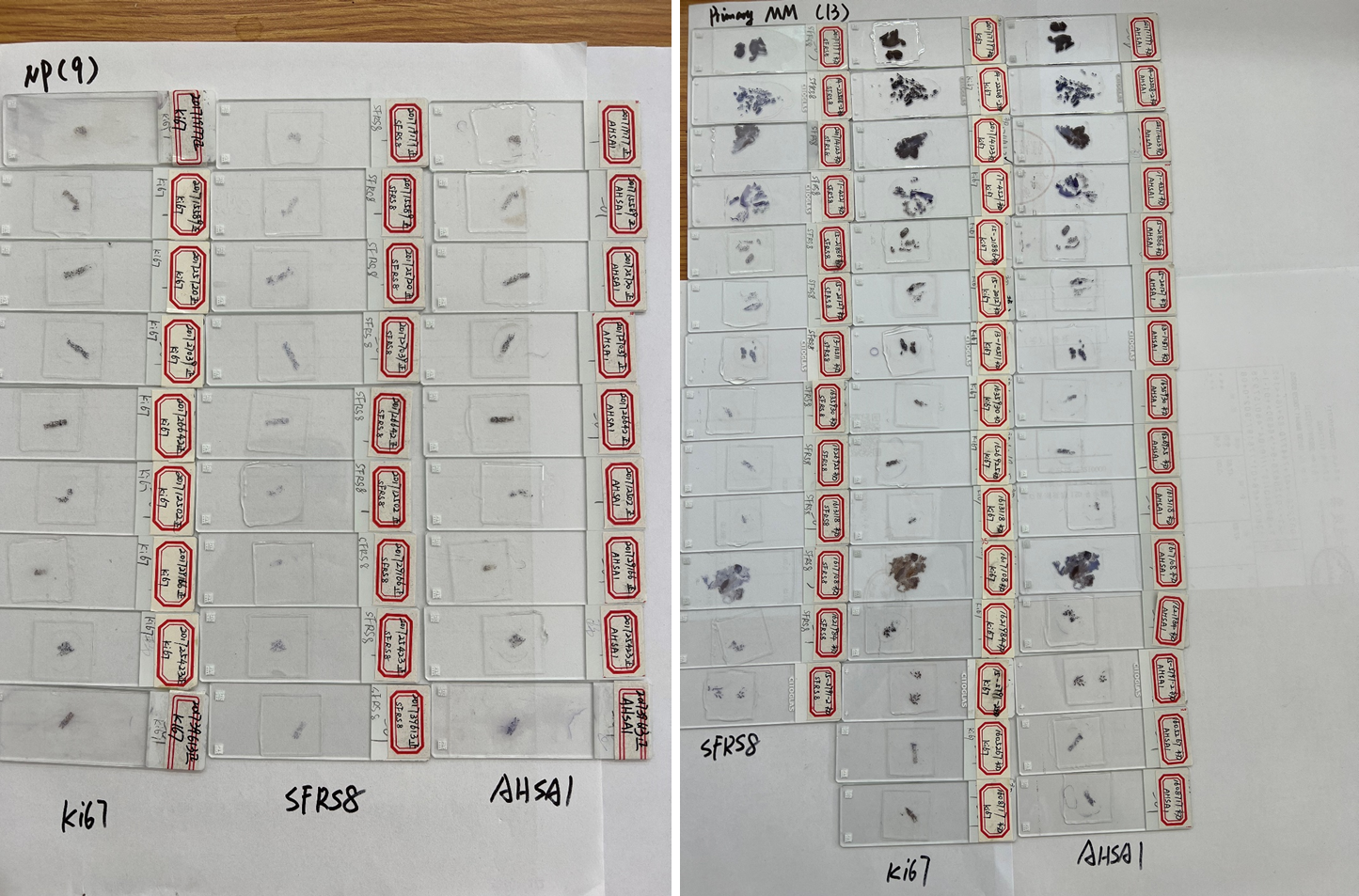


**The patient tissue sections for Immunohistochemistry analysis**


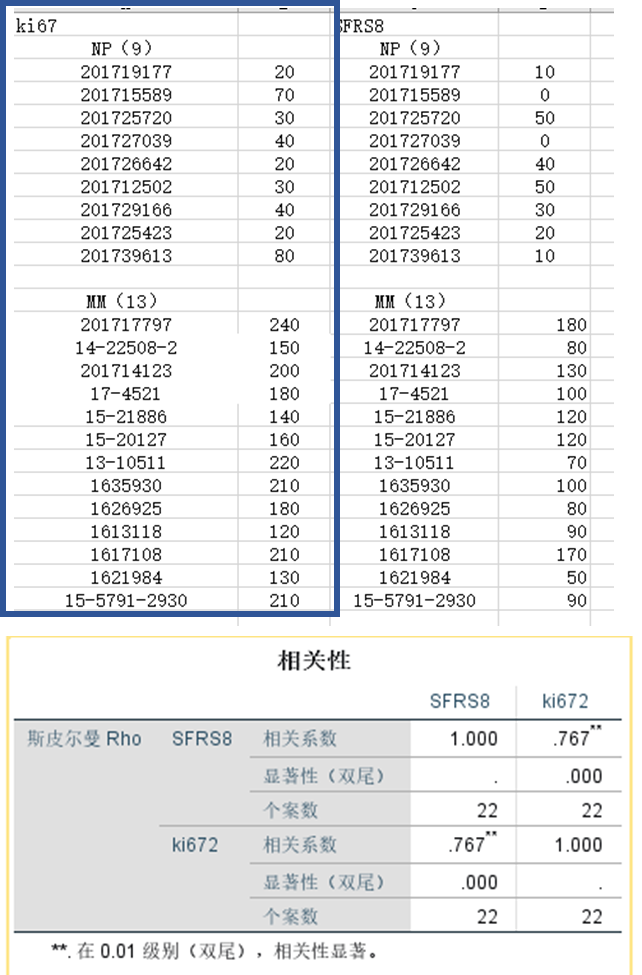


**The correlation analysis between SFRS8 and Ki67**

According to the original data, we re-drafted the correct Figure 1C shown below.


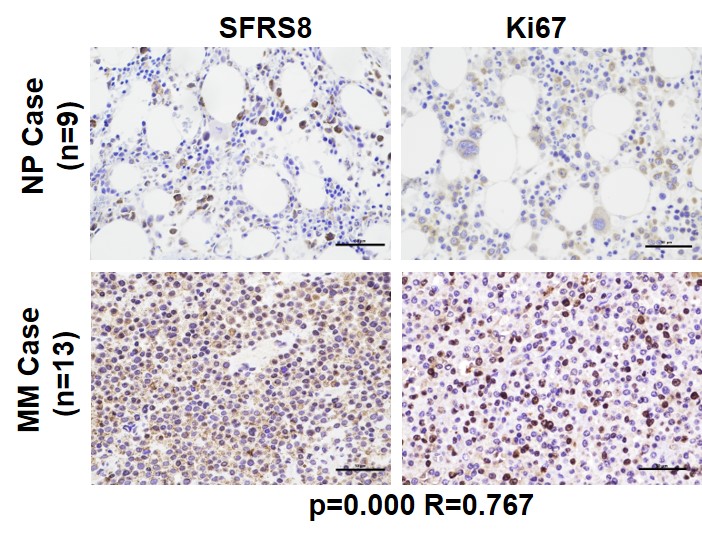


**Corrected Figure 1C**

The reason for replacement of the images is that the represented images of immunohistochemistry analysis for SFRS8 project are misemployed the same images of AHSA1 project (published in J Exp Clin Cancer Res). We used the same batch of patient samples for different projects, which could be proved by the original records. Since two patient tissue sections had run out (1605267 and 1608717) in AHA1 project, the number of MM patients in two projects was different (15 of AHA1 & 13 of SFRS8). We misemployed the same images of Ki67 staining in two articles by error. Since we believe that the correction does not have any effect on the results or conclusions of the paper, we ask your kind consideration for our correction request.
