# Supplementary material for: Correction to“Splicing factor arginine/serine‐rich 8 promotes multiple myeloma malignancy and bone lesion through alternative splicing of CACYBP and exosome‐based cellular communication”
Source: Clin Transl Med. 2023 May 25;13(5):e1282. doi: 10.1002/ctm2.1282 (PMC10212051; doi:10.1002/ctm2.1282)
Supplement: Supplementary file 3 — Supporting Information [file CTM2-13-e1282-s001.docx]

**Supplementary file 2 for the instruction and original data of Figure S3**

We identified one error in upper panel of Figure S3 (ARP1 cell) as following:

Two bands, (1^st^ row, 1^st^ column) and (1^st^ row, 2^nd^ column), were represented by incorrect images.


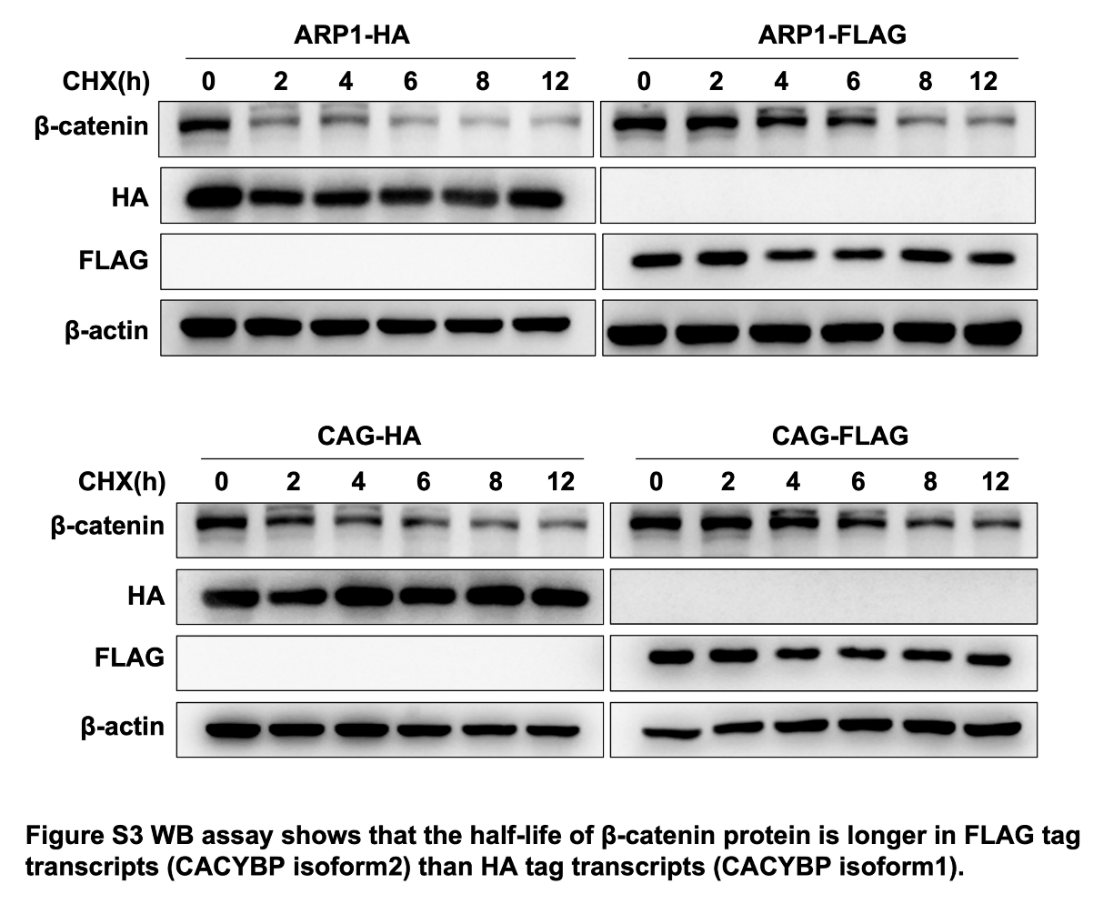


**The upper panel of Figure S3 (ARP1 cell) in the published manuscript**

We collected all the original western blot images as follows:
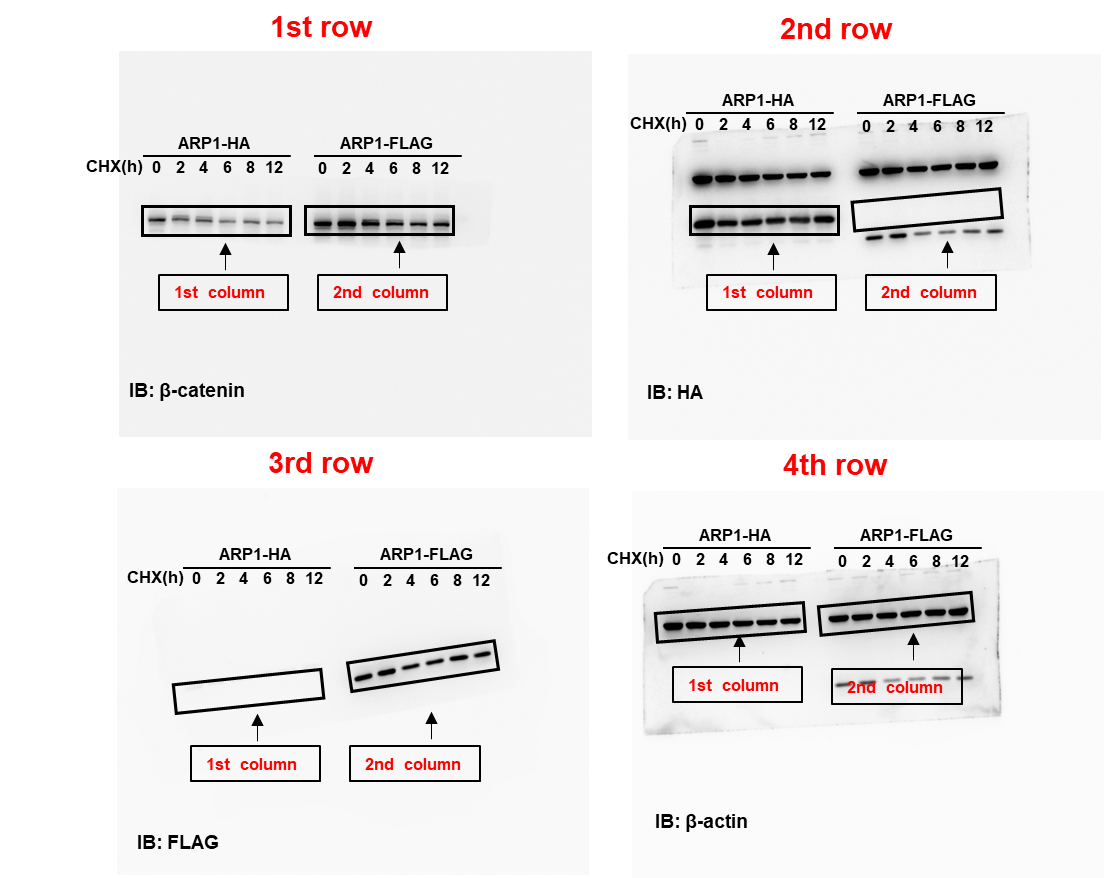


According to the original data, we re-drafted the correct **upper panel of Figure S3** shown below.


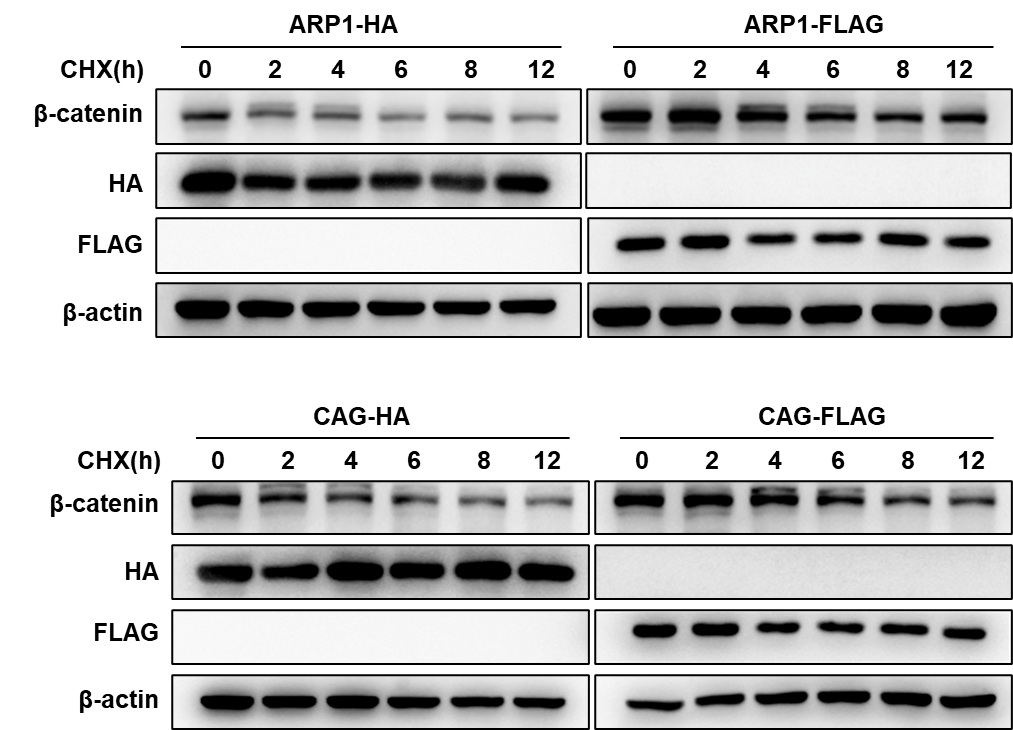


**The upper panel of Figure S3 (ARP1 cell)**

We found that the misused figure was from the data of another project actually, which was placed inappropriately here. We have found the exact bands including the repeated experimental results. Since we believe that the correction does not have any effect on the results or conclusions of the paper, we ask your kind consideration for our correction request.
